# *In vitro* and *in vivo* expansion of CD33/HBG promoter-edited HSPCs with Mylotarg

**DOI:** 10.1016/j.omtm.2024.101343

**Published:** 2024-09-21

**Authors:** Aphrodite Georgakopoulou, Chang Li, Hans-Peter Kiem, André Lieber

**Affiliations:** 1 University of Washington, Department of Medicine, Division of Medical Genetics, Seattle, WA 98195, USA; 2 Fred Hutchinson Cancer Center, Seattle, WA, USA

**Keywords:** *in vivo* HSC gene therapy, CD33, gemtuzumab ozogamicin, GO, HbF reactivation, adenovirus vector, hemoglobinopathies

## Abstract

We developed an *in vivo* HSC gene therapy approach that consists of HSC mobilization and intravenous injection of HSC-tropic HDAd vectors. To achieve therapeutically relevant numbers of corrected cells, we incorporated *in vivo* expansion of transduced cells. We used an HDAd vector for a multiplex adenine base editing approach to (1) remove the region within CD33 that is recognized by gemtuzumab ozogamicin (GO) (Mylotarg), and (2) create therapeutic edits within the HBG1/2 promoters to reactivate γ-globin/HbF. *In vitro* studies with HDAd-transduced human CD34^+^ cells showed editing of both targeted sites and a 2- to 3-fold GO-mediated expansion of edited erythroid/myeloid progenitors. After erythroid *in vitro* differentiation, up to 40% of erythrocytes were HbF positive. For *in vivo* studies, mice were transplanted with human CD34^+^ cells. After engraftment, HSCs were mobilized with G-CSF/AMD3100 followed by an intravenous HDAd injection and GO-mediated *in vivo* selection. Two months later, editing in human cells within the bone marrow was significantly higher in GO-treated mice. The percentage of HbF^+^ human erythroid cells was 2.5-fold greater compared with untreated mice. These data indicate that *in vivo* GO selection can increase edited erythroid cells.

## Graphical abstract

**Figure:**
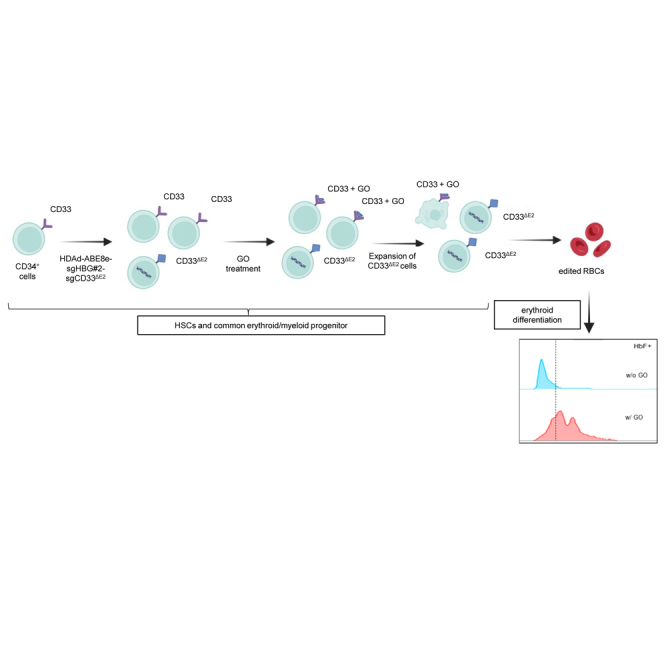

## Teaser

Lieber and colleagues describe a novel HDAd-based *in vivo* HSC transduction and multiplex CD33/HBG promoter editing approach that can significantly increase erythrocytes with re-activated γ-globin expression.

## Introduction

We have previously developed a minimally invasive and readily translatable approach for *in vivo* HSC gene therapy of hemoglobinopathies. It involves mobilization of HSCs from the bone marrow into the peripheral blood by G-CSF/AMD3100 and *in vivo* transduction of HSCs by helper-dependent adenovirus (HDAd) vectors that target receptors present on HSCs, e.g., CD46.^1^^,^^2^^,^^3^^,^^4^^,^^5^ To achieve therapeutically relevant gene correction levels, we have used *in vivo* expansion of transduced HSCs. One such approach is based on a mutant O^6^-methylguanine-DNA methyltransferase (mgmt^P140K^) gene that confers resistance to O^6^-benzylguanine (O^6^BG)/bis-chloroethylnitrosourea (BCNU/carmustine). We have studied this in mice^6^ and non-human primates (NHPs)^4^^,^^7^ using three to four injections of O^6^BG and BCNU at doses that are lower than used for chemotherapy of cancer. In studies toward *in vivo* HSC gene therapy of hemoglobinopathies, we used O^6^BG/BCNU selection in combination with *in vivo* γ-globin gene addition,^2^^,^^4^ *in vivo* base editing to reactivate γ-globin,^8^ and *in vivo* prime editing to correct the sickle cell disease (SCD) mutation.^5^

However, for non-oncological applications, especially for genetic diseases, an *in vivo* selection approach that avoids chemotherapy of genotoxic agents would be preferable. We have therefore been exploring a series of *in vivo* HSC selection/expansion methods that do not require chemotherapy drugs. One of these strategies is based on CD33 editing in combination with a CD33-specific antibody-drug conjugate.

CD33 is overexpressed in a number of hematological malignancies, most notably AML, and has been used as a target for immunotherapy. Among CD33-specific immunotoxins is the antibody-drug conjugate gemtuzumab ozogamicin (GO) (Mylotarg). CD33 is also expressed on HSCs and on common erythroid/myeloid progenitor cells. It is lost during erythroid differentiation (ED)^9^; proerythroblasts are already CD33 negative. After differentiation and exit from the bone marrow, CD33 is present mostly on myeloid cells in peripheral blood, e.g., neutrophils.

CD33 CRISPR-Cas9 editing has been reported either via CD33 gene knockout^10^^,^^11^ or by mimicking a naturally occurring phenotype using a dual guide excision approach to remove exon 2 of CD33. Exon 2 encodes the domain recognized by GO and most other current anti-CD33 targeting moieties. Both approaches, when applied to CD34^+^ cells, did not affect long-term multilineage engraftment,^12^ and it is thought that CD33 is dispensable for hematopoiesis. This and the availability of GO sparked the interest in using CD33 as a target for expansion of genetically modified HSCs for gene therapy. The approach would involve the knockout of CD33 or E2 exon removal by genome editors and the selection of modified HSPCs by GO in combination with the introduction of a therapeutic module, e.g., a genome editor for re-activation of γ-globin. The feasibility of such an approach was demonstrated by Humbert et al.^13^ and, recently, by Borot et al.^14^

Most CD33 editing studies utilized CRISPR-Cas9 that creates double-strand DNA breaks (DSBs) and unpredictable indels. It also becomes increasingly evident that DSBs induced by CRISPR-Cas9 can induce p53-mediated apoptosis and large genomic rearrangements.^15^^,^^16^^,^^17^ In contrast, base editors are capable of introducing precise cytidine or adenine substitutions with minimal occurrence of DSBs and indels at the target site, which greatly reduces the risk of genotoxicity. We have recently used an adenine base editor (ABE8e) that targets a γ-globin repressor (BCL11A) site within the γ-globin promoter genes (HBG1 and HBG2).^8^^,^^18^ Conversion of the target adenine at position −113 to a guanine destroys the BCL11A binding site, and bystander editing of the next adenine at position −116 creates a binding site for the transcriptional activator TAL1. These edits result in reactivation of γ-globin expression reaching therapeutically relevant levels (20% of adult β-globin).

In this study, we used ABE8e in combination with a CD33-specific sgRNA to mediate the skipping of CD33 exon 2 and the HBG1/2-specific sgRNA mentioned above. We hypothesized that editing of CD33 and GO selection at the stage of HSCs and/or erythroid/myeloid progenitors would increase the number of edited erythroid cells that would express γ-globin. We tested this hypothesis *in vitro* using CD34^+^ cells from healthy donors, as well as in humanized mice after *in vivo* HSC transduction and GO selection.

## Results

### Validation of HDAd-ABE8e-sgHBG#2-sgCD33^***Δ***E2^ in ML-1 cells

**Figure 1:**
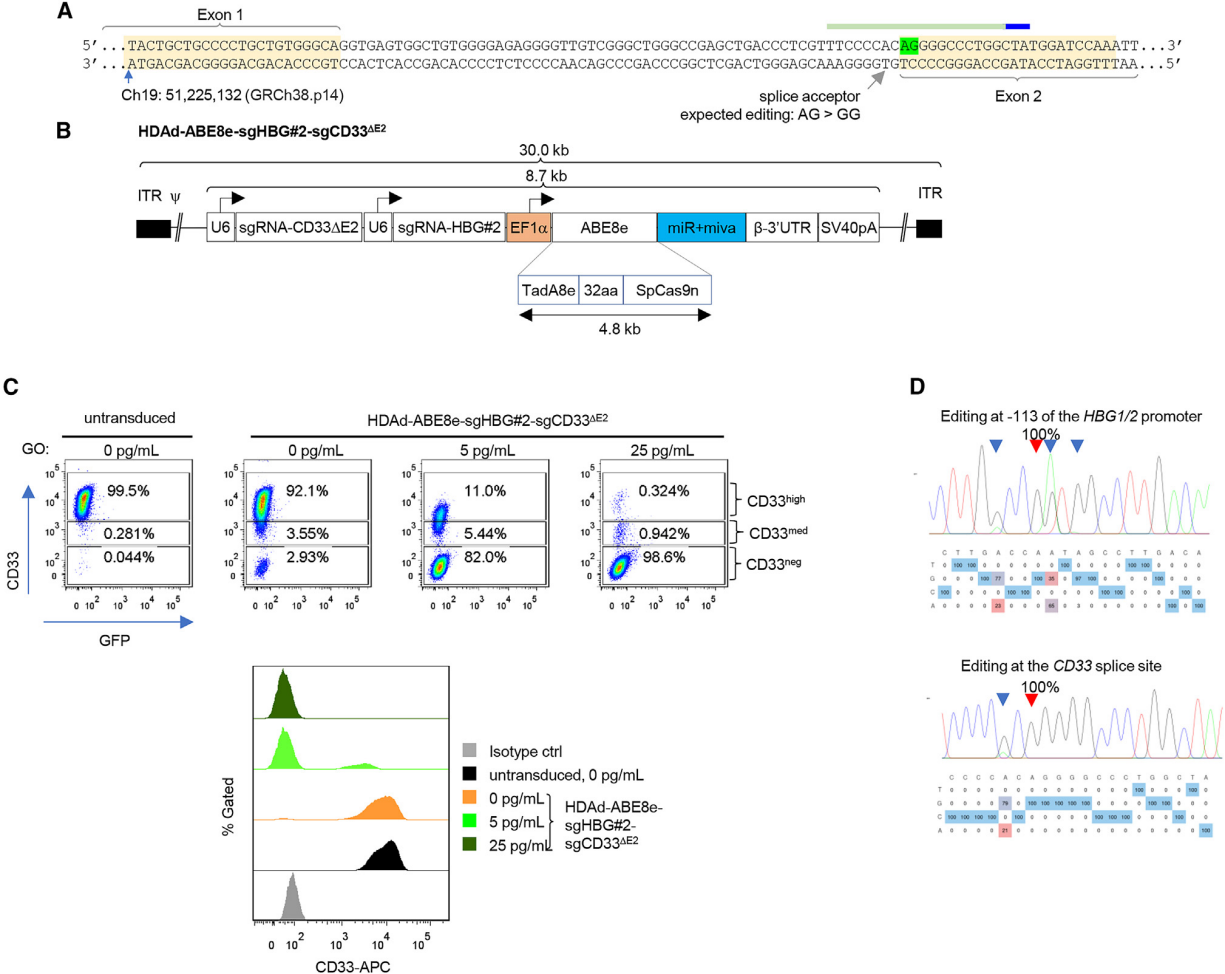
HDAd-ABE8e-sgHBG#2-sgCD33^ΔE2^ vector design and transduction and GO selection studies in the ML-1 cell line (A) CD33 target site and sgCD33^ΔE2^ RNA. The goal is to destroy the “AG” splice acceptor site (labeled in green) by converting into a GG by ABE8e base editing. The corresponding sgRNA is shown above the CD33 sequence with the PAM region labeled in blue. The coordinate of the starting nucleotide of the listed sequence is based on the GRCh38.p14 reference sequence. (B) Vector structure. The vector is based on the CD46-targeting HDAd5/35++ capsid. ABE8e expression is under the control of the strong, ubiquitously active EF1α promoter, while the two gRNAs for the CD33 splice acceptor site and the −113 HBG1/2 site are under the control of pol III U6 promoter. A microRNA responsive element (miR) was embedded in the 3′ human β-globin UTR to minimize toxicity to producer cells by specifically downregulating ABE8e expression in 116 cells. bGHpA, bovine growth hormone polyadenylation sequence; SV40pA, simian virus 40 polyadenylation signal; TadA, adenosine deaminase; ITR, inverted terminal repeat; ψ, packaging signal. The sizes of TadA-SpCas9n, whole transgene and viral DNA are indicated in kilobases. (C) GO selection of HDAd-ABE8e-sgHBG#2-sgCD33^ΔE2^-transduced ML-1 cells. Cells were untransduced (first panels) or transduced at an MOI of 2,000 vp/cell. On day 3 after transduction, GO was added at 0, 5, and 25 pg/mL and, 7 and 14 days later, cells were subjected to flow cytometry for CD33. Figure S2 shows that cells with medium CD33 expression had only one CD33 allele edited, while CD33-negative cells had two edited alleles (and were therefore called CD33^ΔE2^). Upper panel: data shown as dot plots. Lower panel: data shown as stacked histograms. An isotype control was included by staining the untransduced cells with a matching isotype control antibody conjugated with APC. (D) Sanger sequencing results of target sites in transduced ML-1 cells at day 14 after selection with 25 pg/mL GO. Upper panel: editing within the HBG1/2 promoter. The red triangle indicates the target adenine at −113 (100% A>G conversion). The blue triangles mark bystander editing at −110 and −116 adenines. Lower panel: CD33 splice acceptor target site editing (A_7_ > G) (red triangle). The blue triangle marks the editing of a downstream adenine within the intron (A_5_ > G). Editing before selection (14 days after transduction) was also measured. 52% of editing was detected at the target sites of the HBG1/2 promoter.

The ABE8e sgRNA (sgCD33^ΔE2^) for skipping the exon 2 of CD33 was described and validated previously.^14^ It targets the adenine within the splice acceptor site and converts it into a guanine (Figure 1A). The ABE8e sgRNA targeting the −113 position within the HBG1/2 promoter (sgHBG#2) was successfully used in HDAd vectors before.^8^^,^^18^ Here, both sgRNAs were combined into one HDAd vector for multiplex editing (Figure 1B). The resulting vector (HDAd-ABE8e-sgHBG#2-sgCD33^ΔE2^), after transduction, remains episomal and transiently expresses sgRNAs and ABE8e. Vector genomes are lost during cell division; however, the genomic edits are maintained in all progeny cells. The vector was first tested in ML-1 cells, a CD33^+^ myeloid cell line isolated from a patient with AML. ML-1 cells can be efficiently transduced with HDAd5/35++ vectors at MOIs as low as 62 vp/cell (Figure S1A). Flow cytometry with a CD33 exon 2-specific antibody showed that transduction with HDAd-ABE8e-sgHBG#2-sgCD33^ΔE2^ at an MOI of 2,000 vp per cell without GO selection resulted in ∼1.5% of CD33^ΔE2^ cells at day 7 (Figure S1B). Incubation with GO after transduction mediated expansion of CD33^ΔE2^ cells in a GO dose-dependent manner (Figure 1C). At a concentration of 25 pg/mL GO the percentages of CD33^ΔE2^ cells were 59.3% and 98.6% at days 7 and 14 after transduction, respectively. Sanger sequencing of target sites demonstrated that this was due to efficient editing. One hundred percent of the −113 target adenine within the HBG1/2 promoter showed an A > G conversion (Figure 1D). Bystander editing of the −110 and −116 adenines was also observed. Transduction of ML-1 cells with HDAd-ABE8e-sgHBG#2-sgCD33^ΔE2^ and GO selection also resulted in 100% editing of the CD33 adenine target site (A_7_ > G) with 79% editing of a bystander adenine site (A_5_ > G), which located in the first intron and therefore should not affect the CD33 coding sequence. The exact consequence of this bystander editing remains to be investigated in future studies.

Analysis of target site editing in clones derived from single cells by next-generation sequencing (NGS) indicates that, in CD33^neg^ clones, all edits are bi-allelic (Figure S2). No target site editing was detected of CD33^high^ clones. CD33^med^ had a mixture of clones with mono- and bi-allelic edits.

The data in ML-1 cells show that HDAd-ABE8e-sgHBG#2-sgCD33^ΔE2^ mediates editing at both target sites and that GO selection enriches CD33^ΔE2^ cells to nearly 100%.

### Evaluation of HDAd-ABE8e-sgHBG#2-sgCD33^ΔE2^ efficiency in CD34^+^ cells

**Figure 2:**
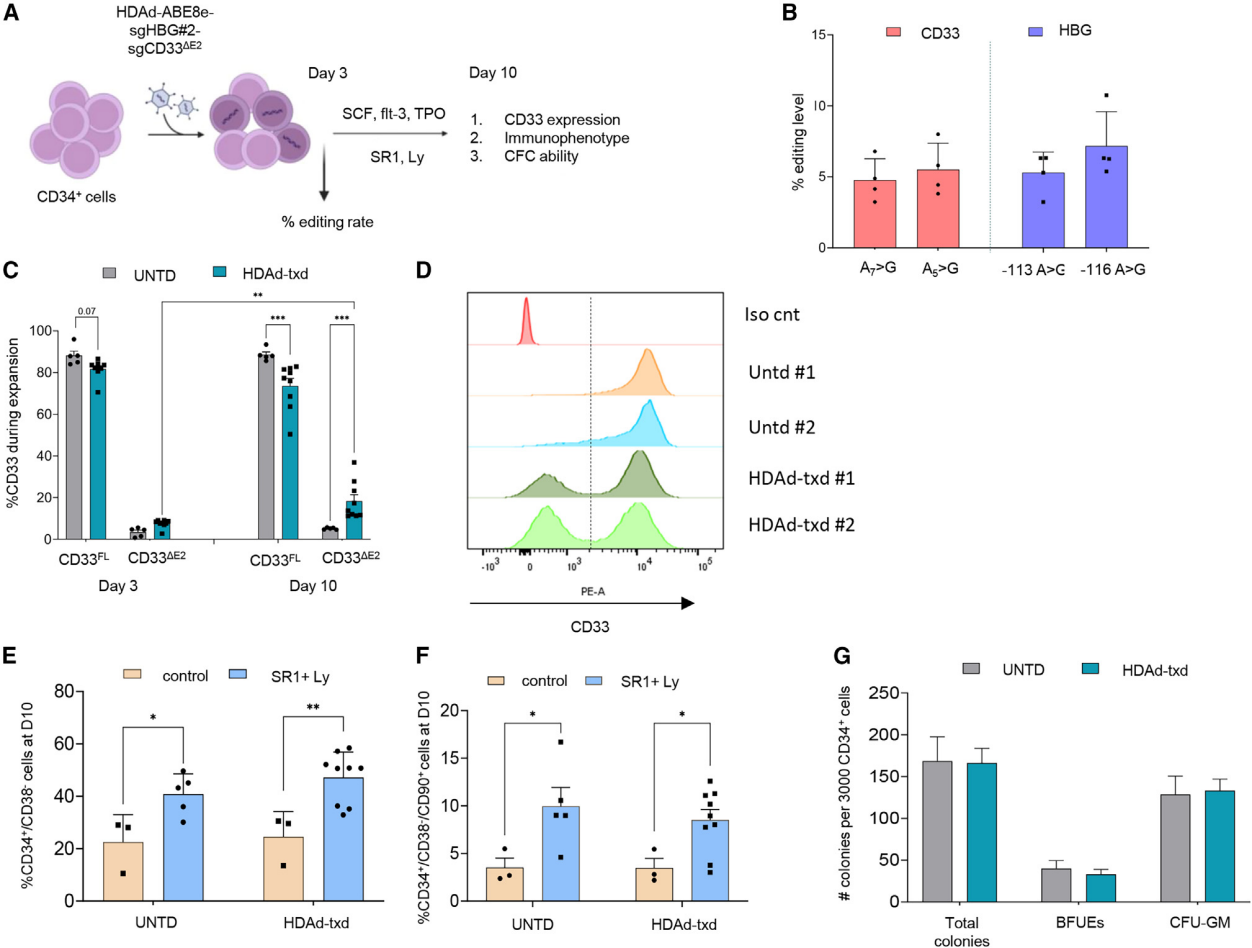
Evaluation of HDAd-ABE8e-sgHBG#2-sgCD33^ΔE2^ transduction in CD34^+^ cells (A) Experimental procedure. CD34^+^ cells from healthy donors (*N* = 4 CD34^+^ cells donors, in duplicate in the transduced group) were cultured in medium supplemented with low cytokines and a combination of the small molecules, SR1 and Ly, to preserve their stemness during the culture. After recovery from cryopreservation, the cells were transduced with the HDAd-ABE8e-sgHBG#2-sgCD33^ΔE2^ vector at an MOI of 4,000 vp/cell and cultured for a total of 10 days. (B) Percentage of target base conversion by HDAd-ABE8e-sgHBG#2-sgCD33^ΔE2^ at both CD33 and HBG sites 72 h post transduction (*N* = 4). NGS data were analyzed by CRISPResso2. (C) Expression of CD33 3 and 10 days post transduction compared with untransduced CD34^+^ cells. CD33^FL^ is the unedited CD33 version (*N* = 4 CD34^+^ cell donors, in duplicate in the transduced group). CD33^ΔE2^ is the exon 2 deleted version. (D) Representative histograms showing the percentage of CD33 levels on day 10. (Ε) Frequency of CD34^+^/CD38^–^ cells on day 10 (*N* = 4 CD34^+^ cells donors, in duplicate in the transduced group). (F) Frequency of CD34^+^/CD38^–^/CD90^+^ cells on day 10 (*N* = 4 CD34^+^ cell donors, in duplicate in the transduced group). (G) Number of total erythroid (BFU-Es), and myeloid (CFU-GM) colonies per 3,000 plated CD34^+^ cells (*N* = 4). Data are shown as means ± SEM. ∗∗∗*p* ≤ 0.001, ∗∗*p* ≤ 0.01, ∗*p* ≤ 0.05 (two-way ANOVA with Bonferroni correction).

After recovery from cryopreservation, >98% CD34^+^ cells isolated from peripheral blood of G-CSF-mobilized donors expressed CD33, indicating that they were at the stage of hematopoietic stem and erythroid/myeloid progenitor cells (Figure S3A). The CD33 expression pattern was maintained for 10 days by incubation in culture medium supplemented with the small molecules StemReginin1 (SR1) and Ly2228820 (Ly), which are known to maintain the stemness of HSCs in culture over time. In our study, this is reflected by the presence of CD33 in >90% of CD34^+^/CD38^–^/CD90^+^/CD45RA^−^/CD49f^+^ cells, a subpopulation that is considered to be enriched for primitive, long-term repopulating HSCs (LT-HSCs) (Figure S3). We used CD34^+^ cells from four healthy donors for transduction with HDAd-ABE8e-sgHBG#2-sgCD3^ΔE2^ at an MOI of 4,000 vp/cell (Figure 2A), an MOI that in previous studies resulted in the transduction of more than 50% of CD34^+^ cells.^5^^,^^8^^,^^18^ Transduced cells were then cultured in SR1+Ly1 peptide medium for a total of 10 days to allow for the accumulation of target site edits^8^^,^^18^ while maintaining the cells at the stage of erythroid/myeloid progenitors. This was important because we aimed to expand edited erythroid progenitor cells by GO.

At 3 day, editing rates at the HBG and CD33 target sites were 5.29% ± 0.73% and 5.51% ± 0.92%, respectively, as measured by NGS (Figure 2B). Bystander editing at the intronic CD33 adenine (A_5_ > G)) site and the −116 HBG1/2 promoter adenine was 7.17% ± 1.2% and 4.76% ± 0.75%, respectively. CD33 flow cytometry performed at day 3 and day 10 post HDAd transduction showed a significant decrease in the percentage of “full-length” CD33^FL^ cells (UNTD vs. HDAd-txd: 88.6% ± 1.37% vs. 73.5% ± 3.6%, *p* = 0.0004), concomitantly with a significant increase of CD33^ΔE2^ cells at day 10 (UNTD vs. HDAd-txd: 5.1% ± 0.21% vs. 18.33% ± 3%, *p* = 0.0021) (Figures 2C and 2D). Importantly, the percentage of “full-length” CD33^FL^ cells was significantly lower 10 days post transduction within the primitive subpopulations of HSPCs (CD34^+^CD38^−^CD90^+^CD45RA^−^CD49f^+^) (Figures S3C–S3E). To assess whether HDAd-ABE8e-sgHBG#2-sgCD33^ΔE2^ transduction affects the phenotype or function of HSPCs, we performed flow cytometry analyses and subjected cells to progenitor colony-forming unit assays. HDAd transduction and target site editing did not alter the frequency of more primitive hematopoietic stem cells (CD34^+^/CD38^–^ and CD34^+^/CD38^–^/CD90^+^) (Figures 2E and 2F), nor their multilineage colony-forming potential (Figure 2G) when compared with untransduced cells.

This study shows that HDAd-ABE8e-sgHBG#2-sgCD33^ΔE2^ mediates editing of both target sites in CD34^+^ cells, which results in the formation of CD33^ΔE2^ cells by day 10 under culture conditions that maintain CD34^+^ cells in an LT-HSC stage (Figure S3C), thus creating a basis for expansion of erythroid cells by GO. Notably, the transduction rate of CD34^+^ cells (and hence the editing rate) depend on the density of the HDAd5/35++ target receptor CD46, which can vary between different donors. CD34^+^ transduction and editing rates are therefore lower compared with transformed cell lines (e.g., ML-1).

### Titration of GO for the enrichment of CD33^ΔΕ2^ cells

**Figure 3:**
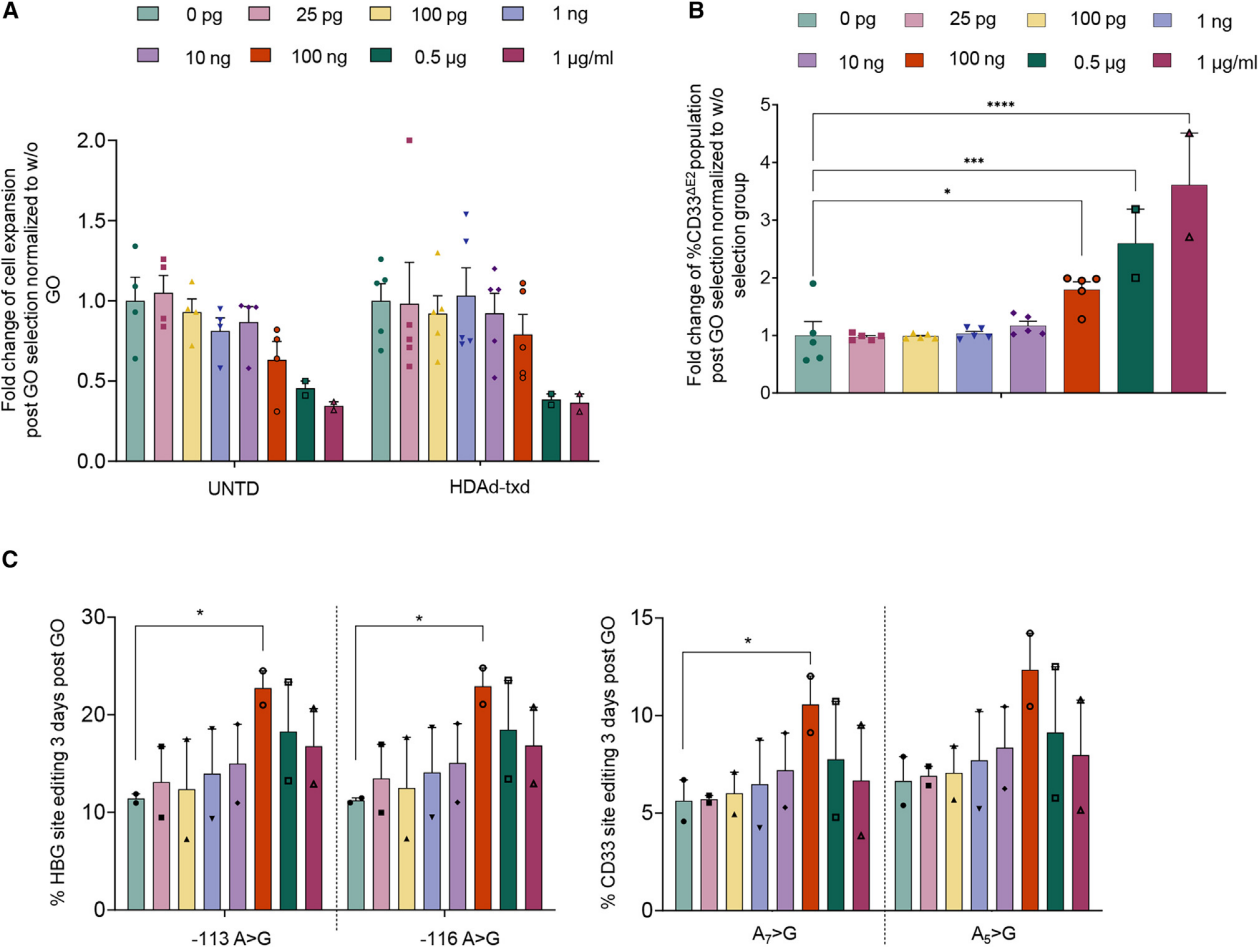
Titration of gemtuzumab ozogamicin in untransduced and transduced CD34^+^ cells (A) Fold change of cell proliferation of untransduced and transduced cells during the gemtuzumab ozogamicin (GO) treatment compared with untreated cells (cells cultured without GO) at different concentrations of the drug. (B) Fold change of CD33^ΔE2^ population of transduced cells after the GO treatment over the untreated transduced cells (cells cultured without GO) at different concentrations of the drug. (C) Editing levels in HBG and CD33 sites post GO selection at different concentrations of the drug, as assessed by NGS. All plots represent data from at least three different donors (some in duplicate). Data are shown as means ± SEM. ∗∗∗∗*p* ≤ 0.0001, ∗∗∗*p* ≤ 0.001, ∗*p* ≤ 0.05 (two-way ANOVA with Bonferroni correction).

**Figure 4:**
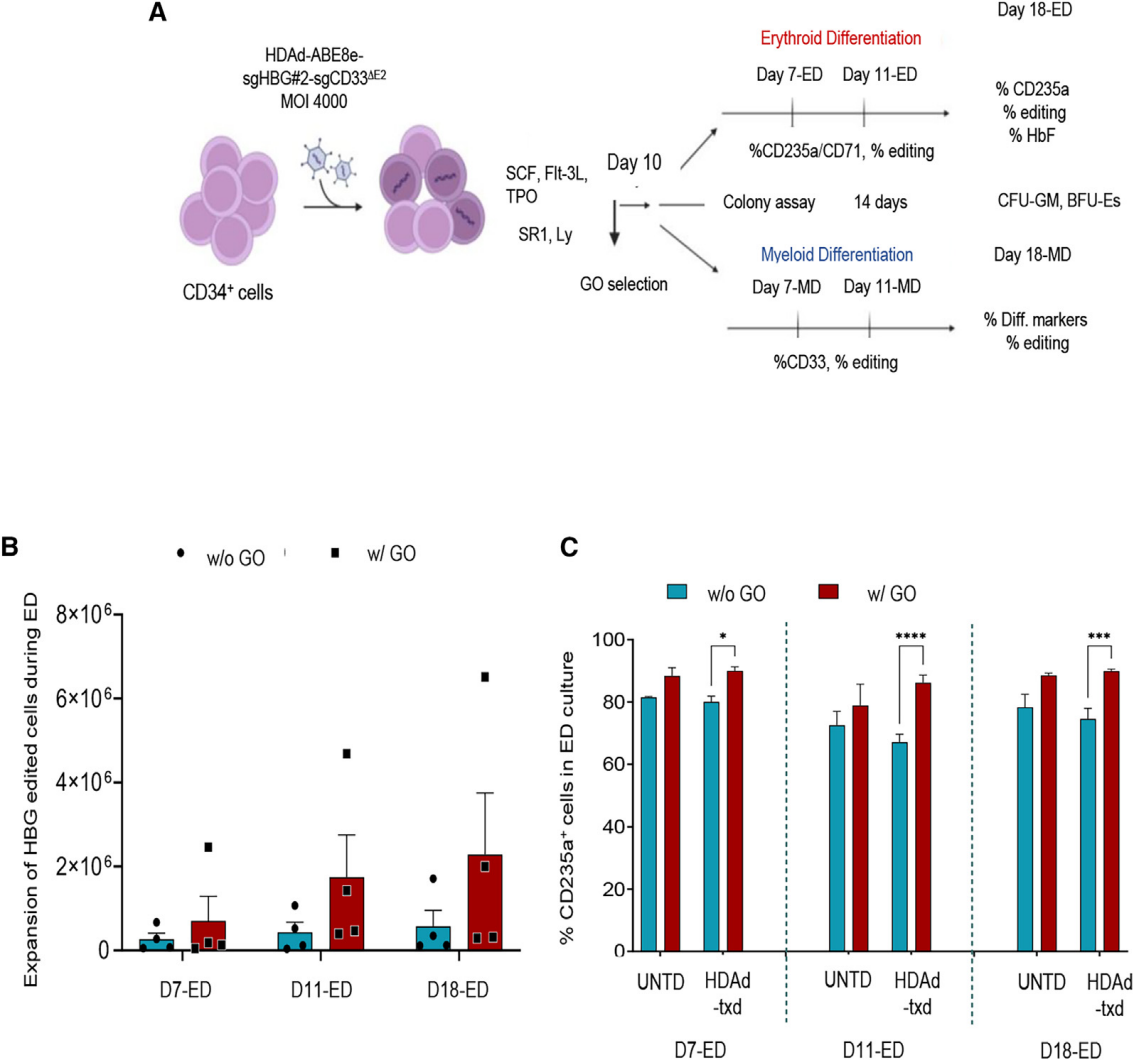
Schematic for *in vitro* differentiation studies and effect of HDAd-ABE8e-sgHBG#2-sgCD33^ΔE2^ transduction and HBG1/2 promoter editing on ED (A) Experimental procedure. Human CD34^+^ cells post GO selection were cultured in methocult for 14 days or in erythroid or myeloid differentiation medium for 18 days. Aliquots of cells were collected at different time points for further analysis. (B and C) Data from ED and GO treatment with 100 ng/mL. (B) Expansion of the HBG1/2-edited erythroid cells (−113 A > G edited cells) during the ED culture. (C) Percentage of CD235a^+^ erythroid cells. All plots represent data from four different donors (some in duplicate). Data are shown as means ± SEM. ∗∗∗∗*p* ≤ 0.0001, ∗∗∗*p* ≤ 0.001, ∗*p* ≤ 0.05 (two-way ANOVA with Bonferroni correction).

On day 10 of CD34^+^ cell culture, we added GO at concentrations ranging from 25 pg/mL to 1 μg/mL and evaluated cell viability, proliferation capacity and editing levels 3 days later (Figure 3). CD34^+^ cells showed higher tolerance to GO than the ML-1 cell line, in which 25 pg/mL of GO resulted in nearly 100% death of non-edited CD33^FL^ cells. Only at GO concentrations higher than 100 ng/mL was a significant reduction in the proliferation rates of untransduced cells observed. At 100 ng/mL of GO, the proliferation rate of HDAd-ABE8e-sgHBG#2-sgCD33^ΔE2^-transduced cells was not significantly affected, suggesting resistance of CD33^ΔE2^ cells to the immunotoxin (Figure 3A). This is supported by flow cytometry analyses that showed a ∼2-fold increase of CD33^ΔE2^ cells in the presence of 100 ng/mL GO (Figure 3B). Notably, the treatment with 100 ng/mL GO, resulted in significant decrease of the CD33^FL^ within the primitive subpopulations of HSPCs (Figure S3C). Even though higher concentrations of GO showed a significant increase of CD33^ΔE2^ cells, these cells were unable to further expand in culture, most likely due to non-target cytotoxicity of the drug. Analysis of editing via NGS on day 3 after addition of GO showed the highest editing rates of both target sites at 100 ng/mL GO, which is consistent with the conclusion about unspecific cytotoxicity, when higher GO concentrations were used (Figure 3C). After treatment with GO at 100 ng/mL, the editing rates at the HBG1/2 and the CD33 sites were ∼20% and 10%, respectively. Based on these data, we decided to use 100 ng/mL GO for further *in vitro* studies with CD34^+^ cells that were subjected to either erythroid or myeloid differentiation (Figure 4A). These data also indicate a relatively narrow window of GO concentrations for selection/expansion of CD33^ΔE2^ cells at the level of HSPCs.

### Expansion of edited erythroid cells during ED and induction of HbF post GO selection

**Figure 5:**
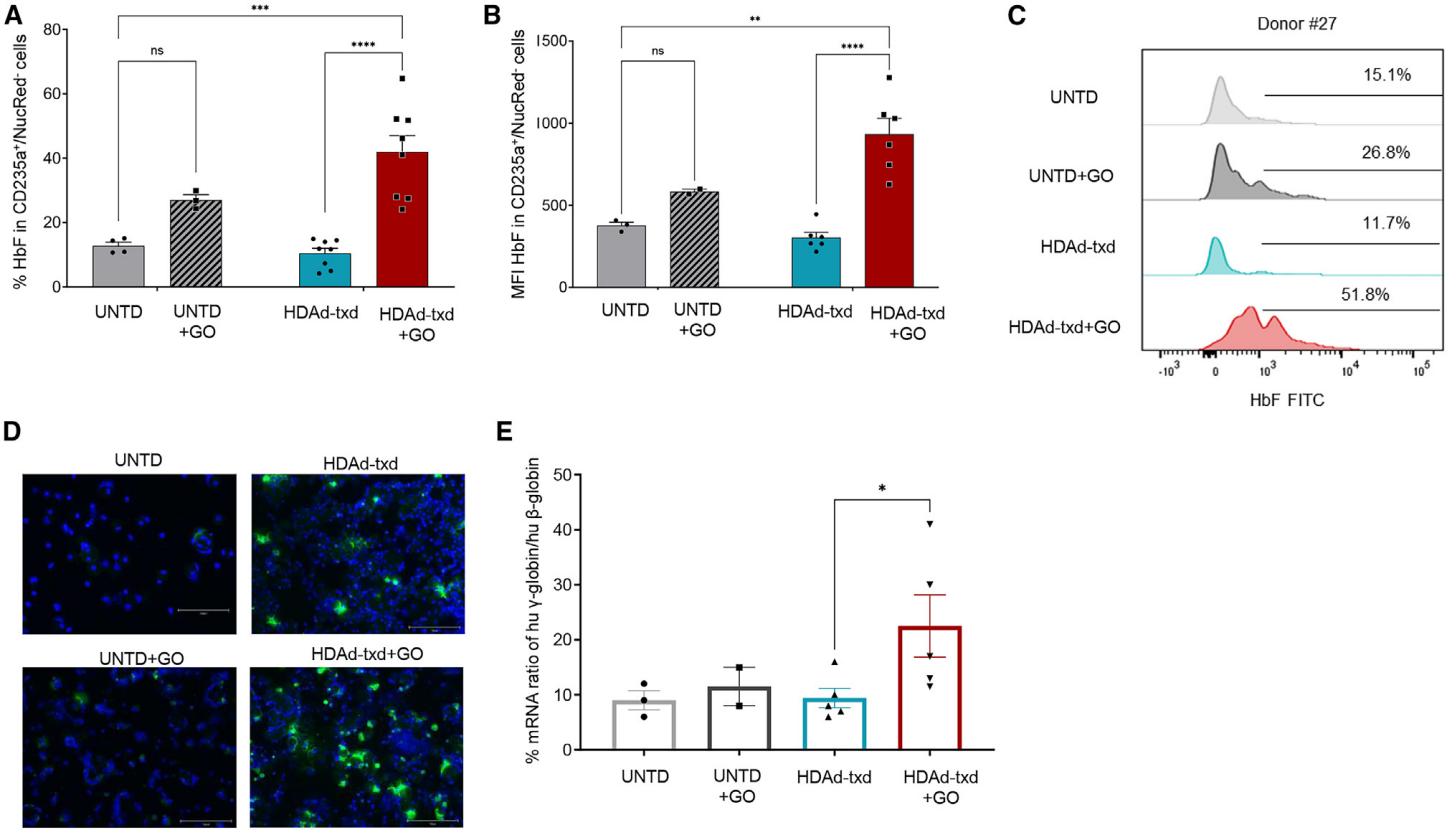
Effect of HDAd-ABE8e-sgHBG#2-sgCD33^ΔE2^ transduction and GO selection on reactivation of HbF (A) HbF expression in enucleated erythroid (CD235^+^/NucRed^−^) cells at the end of ED culture, assayed by flow cytometry. (B) Mean fluorescence intensity of HbF in enucleated erythrocytes at the end of ED. (C) Representative histograms of HbF expression from a representative CD34^+^ cell donor on day 18 of differentiation. (D) Immunofluorescence analysis of HbF on cytospins from cells on day 18 of ED. Green, HbF; blue, nucleus. Scale bar, 150 μm. (E) γ-Globin expression at mRNA level at day 18 of ED, as evaluated by qRT-PCR in comparison with adult β-globin mRNA levels. All plots represent data from four different donors (some in duplicate). Data are shown as means ± SEM. ∗∗∗∗*p* ≤ 0.0001, ∗∗∗*p* ≤ 0.001, ∗∗*p* ≤ 0.01, ∗*p* ≤ 0.05 (two-way ANOVA with Bonferroni correction).

A schematic outline of the *in vitro* differentiation studies is shown in Figure 4A. After HDAd transduction and treatment with GO on day 10, cells were subjected to either erythroid or myeloid differentiation for 18 days. A fraction of cells was plated for progenitor colony assays. Before and during ED, target site editing, cell expansion, and γ-globin re-activation were evaluated. The editing frequency at the HBG1/2 target sites increased during ED, most likely due to an expansion of edited erythroid cells (Figure 4B). The latter is supported by flow cytometry analysis with an antibody recognizing CD235a, an erythroid lineage-specific marker, which showed significantly higher percentages of CD235a^+^ cells in HDAd-transduced, GO-selected settings during ED (Figure 4C). Importantly, a significantly higher percentage of enucleated erythroid (CD235a^+^/NucRed^−^) cells expressed HbF and at higher MFIs compared with control settings at the end of ED (Figures 5A–5C). Increased HbF expression was also visualized by staining of cytospins at the end of the ED culture with a human HbF-specific antibody (Figure 5D). The increased HbF expression was verified by quantification of γ-globin to β-globin mRNA, where an increase of ∼2.5-fold was observed in the GO-selected group (Figure 5E). Furthermore, we observed a higher number of maturing erythrocytes in cytospins made from HDAd transduced and GO-selected cells (Figure S4). This could be explained by the fact that incubation with GO in early stages of the culture (where common erythroid/myeloid progenitors are present), could provide a proliferative advantage of erythroid-committed cells while CD33^FL^ myeloid cells are killed by GO. Notably, flow cytometry data show that GO treatment increases the percentage and MFI of HbF^+^ cells at the end of ED (possibly due to “stress erythropoiesis”). However, this increase was not significant. Furthermore, it was not observed at the level of globin mRNA. The ratio of human γ-globin mRNA to β-globin measured by qRT-PCR was comparable in untransduced cells and cells post GO selection (Figure 5E).

Taken together, these data show GO-mediated selection of erythroid progenitors (as a result of CD33 editing) and expansion of edited erythroid cells. Simultaneous editing at the HBG1/2 sites mediated HbF expression in these cells resulting in about 40% of HbF-positive erythrocytes.

### Stable CD33^ΔΕ2^ population post GO selection during *in vitro* myeloid culture

**Figure 6:**
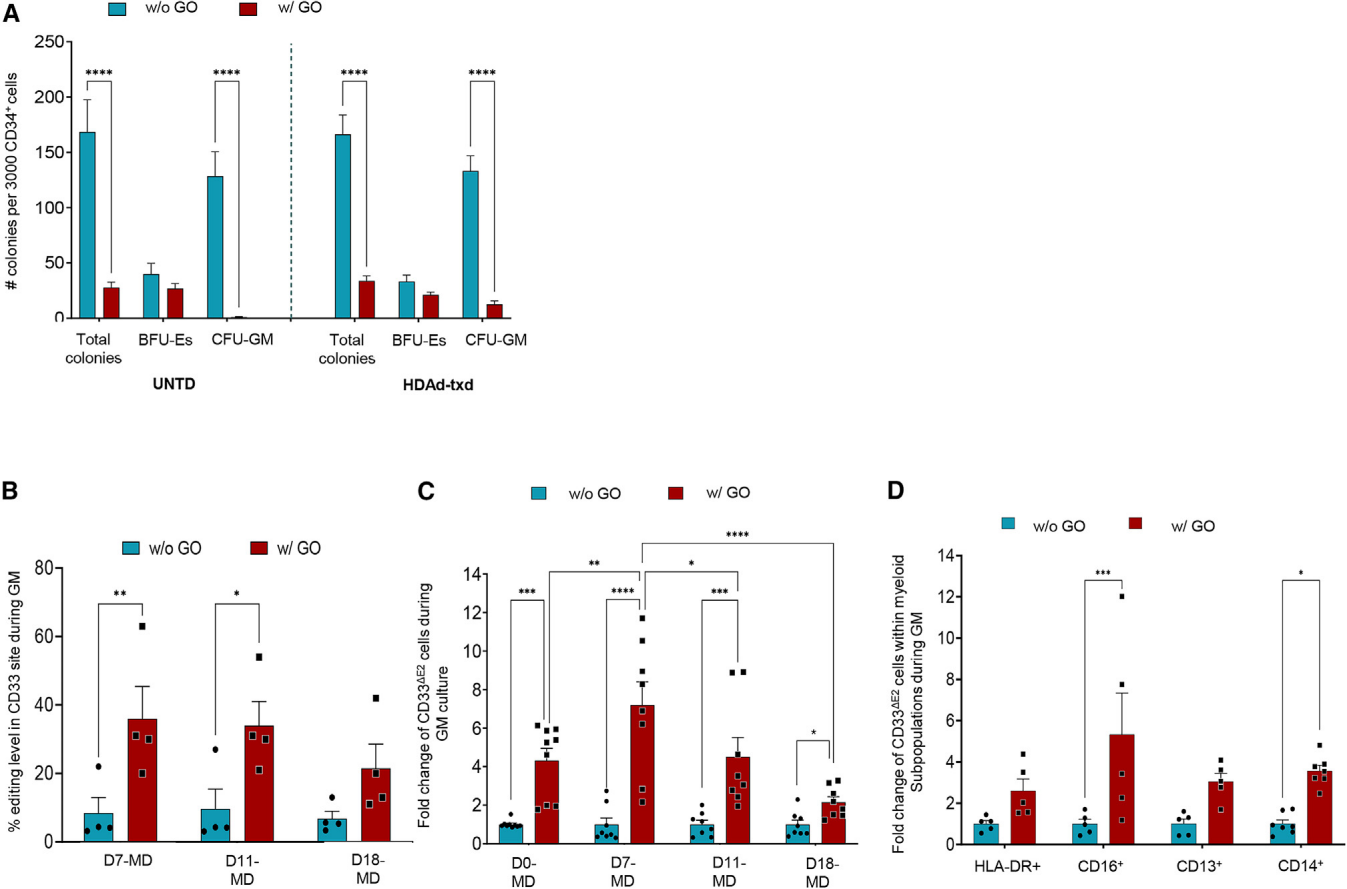
CD33 expression of transduced cells post GO treatment during myeloid differentiation (A) Number of total, erythroid (BFU-Es), and myeloid (CFU-GM) colonies of HDAd transduced and untransduced cells, with or without GO selection. (B) Editing rate of the CD33 target site (A_7_ > G) during myeloid differentiation (*N* = 4), as assessed via NGS. (C) Fold change of CD33^ΔE2^ population during myeloid differentiation (total cells in culture). (D) Fold change of CD33^ΔE2^ subpopulations (HLA-DR^+^, CD13^+^, CD16^+^, and CD14^+^) during myeloid differentiation. All plots represent data from four different donors (some in duplicate). Data are shown as means ± SEM. ∗∗∗∗*p* ≤ 0.0001, ∗∗∗*p* ≤ 0.001, ∗∗*p* ≤ 0.01, ∗*p* ≤ 0.05 (two-way ANOVA with Bonferroni correction).

While the data in erythroid cells are relevant for the treatment of hemoglobinopathies, expansion of edited myeloid cells would be critical for developing new approaches to inhibit HIV. To assess the effect of CD33 editing in myeloid cells, after GO treatment cells were cultured in myeloid differentiation medium, and several myeloid subpopulations were analyzed during the 18-day culture. The effect of GO-mediated killing of CD33^FL^ cells was clearly visible in colony-forming unit (CFU) assays (Figure 6A), where the number of CFU-GM colonies was significantly lower compared with non-selected cells. Notably, the number of CFU-GM colonies from selected cells was higher in HDAd-ABE8e-sgHBG#2-sgCD33^ΔE2^-transduced cells (HDAd-txd). Analysis of cells in myeloid suspension culture also showed overall suppression of proliferation of total myeloid cells by GO (Figures S5A and S5B). Editing rates in the CD33 target site increased ∼3-fold in cells that were treated with GO prior to myeloid differentiation and remained stable during myeloid differentiation, indicating that selection occurred at the level of CD33^+^ progenitors (Figure 6B). This also resulted in an increase of total CD33^ΔE2^ cells in GM culture (Figure 6C). Within myeloid subsets CD33^ΔΕ2^ cells increased ∼3- to 6-fold within HLA-DR cells, ∼10- to 20-fold within CD16 cells, ∼5- to 10-fold within CD13 cells, and ∼4-fold within CD14 cells (Figure 6D). During myeloid culture, no differences were observed in the differentiation and maturation of CD33-edited and GO-selected cells during morphological evaluation of cytospins (Figure S5C).

The data from myeloid cultures indicate that HDAd transduction and GO treatment results in substantial expansion of edited CD33^ΔE2^ myeloid cells including subfractions that are targets for HIV infection.

### *In vivo* transduction/editing followed by *in vivo* GO selection of xenografted human CD34^+^ cells results in high expression of γ-globin in erythroid cells

**Figure 7:**
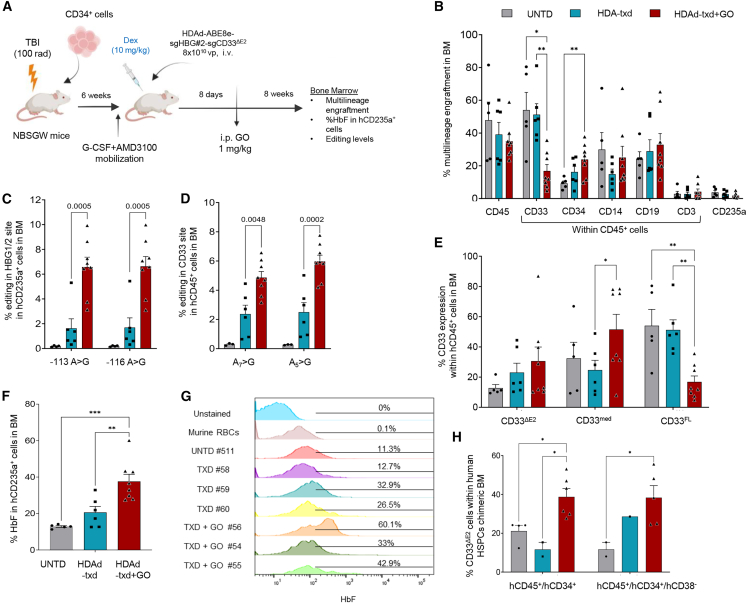
*In vivo* gene editing of human CD34^+^ cell by HDAd-ABE8e-sgHBG#2-sgCD33^ΔE2^ and *in vivo* selection of edited cells by GO in humanized mice (A) Experimental procedure of *in vivo* transduction experiments. In brief, CD34^+^ cells from healthy donors were transplanted in TBI-treated NBSGW mice (*N* = 19). Six weeks post transplantation, CD34^+^ cells were mobilized to the peripheral blood by G-CSF and AMD3100. At the peak of mobilization, the mice were injected i.v. with HDAd-ABE8e-sgHBG#2-sgCD33ΔE2, and 1 week after *in vivo* transduction the mice received i.p. GO at a total dose of 1 mg/kg (*N* = 8) (UNTD group *N* = 5 and HDAd-txd group *N* = 6). Two months later, the mice were euthanized, and their hematopoietic tissues were collected for further analysis. (B) Multilineage reconstitution 2 months after *in vivo* transduction and GO administration measured by flow cytometry with antibodies against human cell surface markers. (C and D) Editing levels in the targeted and bystander adenines of human cells isolated from the chimeric BM in (C) HBG1/2 promoter site and D) CD33 site. (E) Analysis of the percentage CD33 expression within the CD45^+^ cells in BM. (F) HbF expression in human engrafted erythroid cells in the chimeric BM, as measured by flow cytometry. (G) Representative histograms of HbF expression from the engrafted erythrocytes. Murine erythrocytes were used as negative control. (H) Percentage of CD33^ΔΕ2^ cells within human HSPCs in the chimeric bone marrow. Each symbol represents an individual mouse. Data are shown as means ± SEM. ∗∗*p* ≤ 0.01, ∗*p* ≤ 0.05 (two-way ANOVA with Bonferroni correction).

We and others have previously reported that the *in vivo* transduction of human CD34^+^ cells in humanized mice is feasible by HDAd5/35++ vectors after mobilization of human HSPCs from the chimeric bone marrow to the periphery.^19^^,^^20^^,^^21^ In brief, our approach consists of transplantation of CD34^+^ in partially myeloablated NBSGW mice (100 rad TBI) and engraftment of human HSCs (Figure 7A). Six weeks after transplantation, HSCs were mobilized with a 7-day G-CSF/AMD3100 mobilization scheme followed by an intravenous injection of the HDAd-ABE8e-sgHBG#2-sgCD33^ΔE2^ vector at the peak of mobilization. One week post *in vivo* transduction, the animals received intraperitoneal GO at a dose of 1 mg/kg. Eight weeks after the last GO injection, the mice were sacrificed, and the hematopoietic tissues were harvested for analysis. At the time of sacrifice, multilineage engraftment in the bone marrow was evident in all mice. Based on the flow cytometry of human CD45^+^ cells, donor chimerism in the bone marrow was ∼50% in untreated mice, ∼40% in transduced mice, and ∼35% in GO-treated/transduced mice. HDAd-transduced, GO-treated mice showed significantly increased CD34^+^ cells indicating that GO-mediated expansion of edited cells occurred in HSPCs (Figure 7B). It was, however, not carried on to ΗSPC progeny because no significant GO-mediated expansion was seen in lineage-positive cells. HDAd-transduced mice had lower levels of CD14^+^ cells, which however expanded after GO treatment. Measurement of editing levels in human CD45^+^ cells isolated from the chimeric bone marrow by magnetic cell sorting (MACS) showed 6% CD33 editing at the relevant adenine (A2) post GO selection compared with ∼3% in the mice without selection, while HBG editing in the target site (−113A) was ∼6% after selection versus ∼2% in their unselected counterparts (Figures 7C and 7D). Increased editing, most likely, was causative of higher percentage of CD33^ΔE2^ cells in the bone marrow of GO-treated mice, with subsequently lower percentage of CD33^FL^ cells in total CD45^+^ cells (Figure 7E). Importantly, in mice that were transduced with the HDAd vector and subjected to GO selection, 40% of erythroid (CD235a^+^) cells were positive for γ-globin (HbF) (Figures 7F and 7G). The percentage of HbF^+^/CD235a^+^ cells in the bone marrow of mice that did not receive GO was ∼20%. In untreated mice, 10% of human erythroid cells expressed HbF. This high background might be due to incomplete ED of erythroid cells in this mouse model, which is clearly a limitation for the efficacy readout of our approach. Importantly, the significantly higher percentage of HbF^+^ erythroid cells was the result of HBG1/2 site editing and GO-mediated expansion of CD33^ΔE2^ erythroid progenitors. Notably the percentage of CD33^ΔΕ2^ cells was increased within the human HSPCs *in vivo* after the GO treatment (Figure 7H). Regarding the safety of GO administration, no alterations in blood cell counts were found at the end of the study (Figures S6A–S6C) and the hematological parameters did not show any abnormalities (Figure S6B). Also, no differences were observed in the colony-forming unit potential of human CD45^+^ cells isolated from the chimeric bone marrow of the mice between the groups (Figure S6D). Finally, no changes were observed in the weight of the mice (Figure S6E). The overall physical appearance of GO-treated mice did not differ from the untreated mice. The absence of side effects of GO treatment is not surprising because GO does not interact with mouse CD33.

Notably, administration of higher and multiple doses of GO in *in-vivo*-edited humanized mice did not increase the HBG editing levels or the HbF expression but reduced them, suggesting that higher doses of GO are toxic, probably at the level of edited and non-edited erythroid/myeloid progenitors (Figures S7A and S7B). This is in agreement with our *in vitro* studies (see Figure 3).

The *in vivo* data indicate that editing of both sites occurs *in vivo* and that one-time GO treatment can expand the CD33^ΔE2^-HBG1/2-edited erythroid cells resulting in erythrocytes with reactivated γ-globin. The effective GO dose range is, however, very narrow, and the expansion rate is only ∼2.5-fold.

## Discussion

Here we show HDAd-mediated *in vivo* gene therapy using ABE8e base editing to increase fetal hemoglobin and select for edited cells *in vivo*. We demonstrate efficient dual ABE8e base editing to modify CD33 and at the same time edit the γ-globin locus to increase fetal hemoglobin in the NBSGW xenotransplant mouse model. After *in vivo* selection with GO, we see a 2.5-fold increase in HbF^+^ human erythroid cells compared with untreated mice.

We have previously shown the transduction of 15%–20% of mouse HSCs (LSK cells)^6^^,^^22^ and ∼7% of rhesus HSCs (CD34^+^/CD45RA^−^/CD90^–^)^23^ (measured at day 7 after HDAd injection in the bone marrow) with our HDAd *in vivo* gene therapy approach. However, not every transduced HSPC is stably modified with integrated transgenes or permanent genome edits. The integrating system requires co-infection of two vectors, the transposon vector and the SB100× vector for stable expression, which limits the number of stably transduced HSPCs. For genome editors, even though cells are transduced, target site editing requires a certain expression level (or VCN) and target site accessibility, which can depend on the proliferation/differentiation stage of cells. For the correction of hemoglobinopathies, 20% of therapeutically corrected erythroid cells need to be present in the periphery.^24^ We therefore combined our *in vivo* transduction approach with an HSPC selection system that is based on the expression of mgmt^P140K^ and O^6^BG/BCNU. This powerful system can achieve almost 100% of gene marking in peripheral blood cells after *in vivo* HSC transduction/selection.^1^ However, it is based on a genotoxic chemotherapy regimen that cannot be used for the treatment of SCD. A major goal has therefore been the development of an *in vivo* HSC expansion system that would allow for high transgene marking in erythrocytes without the need of chemotherapy drugs. Here, we assessed CD33/GO-based HSPC selection. Our hypothesis was that this approach allows for the expansion of common erythroid/myeloid CD33^+^ progenitors and that multiplex (CD33 and HBG1/2 promoter) editing and GO selection would increase the percentage of HbF-expressing (“corrected”) erythrocytes.

The corresponding HDAd-ABE8e-sgHBG#2-sgCD33^ΔE2^ vector was first tested in the myeloid CD33^+^ cell line ML-1. HDAd transduction and GO selection resulted in nearly 100% CD33^ΔE2^ cells, demonstrating the functionality of the vector and the power of the CD33/GO selection system. The percentage of CD33^ΔE2^ cells increased over time (day 7. 59.3% vs. day 14, 98.6%), which we attribute to a gradual increase of editing of the target site. Notably, the base editor is expressed as long as episomal HDAd genomes are not lost due to cell division. The conclusion that prolonged expression of genome editors increases the editing rate is also based on our previous studies with other base and prime editors.^5^^,^^8^ Considering this, we incubated HDAd-transduced CD34^+^ cells for 10 days in medium that suppresses differentiation before we initiated GO selection. On day 10, editing rates for both the HBG1/2 and CD33 target sites were ∼5%–8%. Incubation with GO increased the percentage of edited target sites to a larger degree in myeloid than in erythroid cells (2-fold vs. 5-fold). Although off-target editing was not evaluated in this study, our previous study with the HBG1/2 ABE8e base editor did not detect editing at top-scored off-target sites.^8^

The increase in HBG1/2 promoter editing after selection with GO led to an increase in reactivation of γ-globin expression. GO treatment of CD34^+^ cells resulted in an increase of HbF also in non-transduced cells, although this effect was not significant and was probably the result of stress erythropoiesis^25^ (i.e., activation of fetal expression patterns) triggered by GO. Importantly, on top of this, γ-globin expression was significantly induced by editing the HBG1/2 as γ-globin mRNA studies indicated (see Figure 5E). A clear effect of GO selection on HbF levels was demonstrated by flow cytometry and immunofluorescence analyses, which showed 10% vs. 40% of HbF^+^ enucleated erythrocytes in transduced cells without and with GO selection, respectively. Analysis of cytospins after ED also indicated that GO treatment increased the number of matured erythrocytes (see Figure S3), indicating improved erythropoiesis, a phenomenon that has to be studied further.

Furthermore, we subjected HDAd-transduced and GO-treated cells to myeloid differentiation and demonstrated GO-mediated expansion of edited myeloid cells (see Figure 6). These data are relevant for HIV prophylaxis/therapy,^26^ for example by multiplex editing resulting in knockout of the CCR5 co-receptor and CD33 editing for GO expansion.

Importantly, the ability to expand both edited erythroid and myeloid cells indicates that the dual editing and GO expansion occurred at the stage of common erythroid/myeloid progenitor cells.

A recent study by Borot et al.^14^ also used multiplex CD33-HBG1/2 editing and demonstrated that *ex vivo* ribonucleoprotein-transfected CD34^+^ cells efficiently engraft and repopulate a complete hematopoietic system in NHPs after transplantation into myeloablated animals. This study also shows that GO treatment enriched for dual edited HSPCs *in vitro*, but the study did not evaluate HbF activation after GO treatment and ED. Importantly, we used the approach *in vivo* in mobilized humanized mice that were intravenously injected with HDAd-ABE8e-sgHBG#2-sgCD33^ΔE2^ and intraperitoneally treated with GO for *in vivo* selection. *In vivo* editing of CD34^+^ cells in the xenotransplantation mouse model resulted in ∼3% editing at both CD33 and HBG sites in human cells localized to the bone marrow, which increased ∼2-fold in mice treated with GO 1 week post *in vivo* transduction. Two months after the GO selection, animals showed significantly lower expression of CD33 and higher expression of HbF in human cells indicating the HSCs and/or early progenitors were edited. The percentage of HbF^+^ human erythroid (CD235a^+^) cells was ∼15%, ∼20%, and >40% in untransduced mice, *in vivo* HDAd-transduced mice, and HDAd-transduced + GO-injected mice, respectively. These data show that GO selection can increase therapeutically modified cells at least 2-fold.

A limitation for the use of the CD33/GO approach *in vivo* is the heterogeneous CD33 density on target cells (see Figure S1) and the narrow window of GO doses that can be used to expand the desired cells. In our studies, this problem became apparent in the relatively modest rate of expansion of corrected cells. Both our *in vitro* and *in vivo* studies showed significant toxicity of high GO doses; *in vitro* the dual-edited CD34^+^ cells were unable to proliferate after treatment with high concentrations of GO, while administration of higher GO doses *in vivo* affected the efficacy of the approach, as reflected by reduced editing rates at the end of study. We speculate that cells with only one edited CD33 allele were killed by higher doses of GO.

Collectively, this proof-of-concept study describes a novel HDAd5/35++-based *in vivo* HSC transduction and multiplex CD33/HBG promoter-editing approach that can significantly increase erythrocytes with re-activated γ-globin expression.

## Materials and methods

### Reagents

For *in vivo* transduction, G-CSF (Neupogen) (Amgen, Thousand Oaks, CA), AMD3100 (MilliporeSigma, Burlington, MA) and dexamethasone sodium phosphate (Fresenius Kabi USA, Lake Zurich, IL) were used. GO (Mylotarg, Pfizer) was used for the *in vivo* selection of CD33-edited cells.

### Construction of HDAd-ABE8e-sgHBG#2-sgCD33^ΔE2^

The CD33 guide sequence 5′-CCCCACAGGGGCCCTGGCTA-3′ was inserted into the *Bbs*I site of pSPgRNA (Addgene, no. 47108), generating pSP-sgCD33. The 0.4 kb U6 promoter-sgCD33 region was amplified from pSP-sgCD33 using primers 5′-TCCGCGGTGGGCGCGCCGAGGGCCTATTTCCCATGATTCC-3′ and 5′-TTAGGATCCGGCGCGCCCACCGCGGAAAAAAAGCACCGAC-3′ and inserted into the *Asc*I site of pBS-ABE8e-sgHBG#2-miR-v3^1^. A pHCA derivative named as pHCAS4-MCS was constructed similarly as for pHCAS5-MCS^2^. pHCA4-MCS has 1.3 kb less stuffer DNA than pHCAS5-MCS to accommodate more transgene insertion. pBS-ABE8e-sgHBG#2-miR-v3 and pHCAS4-MCS were linearized by *PacI* and joined by infusion cloning, resulting in pHCA-ABE8e-sgHBG#2-sgCD33. The final construct was validated by restriction enzyme digestion and whole plasmid sequencing. For rescue and amplification of HDAd5/35++ vectors, we used AdNG163-5/35++, an Ad5/35++ helper vector containing chimeric fibers composed of the Ad5 fiber tail, the Ad35 fiber shaft, and the affinity-enhanced Ad35++ fiber knob. Corresponding pHDAd-ABE8e-sgHBG#2-sgCD33^ΔE2^ plasmids were linearized, rescued with either AdNG163-5/35++, and amplified in 116 cells in 3-L spinner flasks as described in detail elsewhere.^27^ Helper virus contamination levels were found to be less than 0.1%.

### ML-1 cells

ML-1 cells (human leukemia, CVCL_0436) were cultured in RPMI supplemented with 10% FBS, glutamine, and streptomycin/penicillin.

### CD34^+^ cell culture

CD34^+^ cells from mobilized healthy donors were obtained from the Fred Hutchinson Cancer Center, Seattle, WA. The cells were recovered from frozen stocks and incubated for 24 h in serum free medium (Stemspan H3000, STEMCELL Technologies) supplemented with penicillin/streptomycin (Gibco), Flt3 ligand (100 ng/mL), thrombopoietin (100 ng/mL), and stem cell factor (SCF) (100 ng/mL) and the small molecules SR1 (1 μΜ) (Cellagen Technology) and Ly2228820 (Ly, 100 nM) (Selleckchem).^28^^,^^29^^,^^30^ All cytokines were obtained from PeproTech.

CD34^+^ cells were transduced with the HDAd-ABE8e-sgHBG-sgCD33 vector in low attachment plates for 48 h, at a total MOI of 4,000 vp/cell and cultured for an additional 7 days, followed by GO selection, before transfer in ED-, myeloid differentiation-, and methylcellulose-based medium.

Dilutions of freshly dissolved GO (Mylotarg, Pfizer) were used for the selection of CD33-edited cells.

### *In vitro* ED of CD34^+^ cells

Differentiation of transduced and non-transduced CD34^+^ cells into erythroid cells was done based on a three-step protocol developed by Douay et al.^31^ In step 1, cells were cultured at a density of 10^4^ cells/mL for 7 days in a basal medium containing Iscove’s modified Dulbecco’s medium (IMDM), 5% human plasma, glutamine, penicillin/streptomycin, heparin (2 IU/mL), insulin (10 μg/mL), holo-transferrin (330 μg/mL) supplemented with hydrocortisone (1 μM), SCF (100 ng/mL), IL-3 (5 ng/mL), and EPO (3 U/mL). In step 2, cells were cultured at a density of 10^5^ cells/mL for 4 days in the same basal medium, as previously, supplemented with SCF (100 ng/mL) and EPO (3 U/mL). Finally, in step 3, the cells were cultured at a density of 10^6^ cells/mL for 7–10 additional days in the same medium, supplemented only with EPO (3 U/mL).

### *In vitro* myeloid differentiation of CD34^+^ cells

Differentiation of human CD34^+^ cells into myeloid cells was done based on the following protocol: 10^4^ cells/mL were cultured in medium containing IMDM, 5% human plasma, glutamine, penicillin/streptomycin, heparin (2 IU/mL), supplemented with SCF (50 ng/mL), IL-3 (5 ng/mL), GM-CSF (10 ng/mL), and G-CSF (20 ng/mL), for 15–18 days. All cytokines were from PeproTech.

### Cell surface staining/flow cytometry

Staining of ML-1 cells was performed with a set of CD33 antibodies, either specific to the V set domain (P67.6), or to the C2 domain of all CD33 isoforms (HIM3-4), or to the C2 domain only when exon 2 is absent (11D5). Cells that stained negative with the E2-specific antibody were labeled as CD33^ΔE2^. To evaluate the HSC phenotype, post double-editing, cells were washed and stained with the following fluorochrome-conjugated antibodies: CD34-APC, CD38-PE, and CD90-PerCP (BD Biosciences, San Jose, CA). For the assessment of CD33 expression in HSPC subpopulations the following antibodies were used: CD34-APC, CD38-PE-Cy7, CD90-PE, CD45RA-APC-Cy7, CD49f-BV711, and CD33-FITC. For the follow-up of CD34^+^ cell differentiation into erythroid or myeloid cells the following antibodies were used: for erythroid cells: CD235a-PE (BD Biosciences) and CD71 FITC (BD Biosciences); for myeloid cells: CD33-PE (BD Biosciences), CD14-FITC (BD Biosciences), HLA-DR-APC (BioLegend), CD13-APC (BioLegend), and CD16-PerCP (BioLegend). After wash, cells were resuspended in FACS buffer and analyzed using a BD FACSymphony A3 Cell Analyzer (BD Biosciences). Debris was excluded using a forward scatter-area and sideward scatter-area gate. Flow cytometry data were then analyzed using FlowJo (version 10.0.8, FlowJo). A complete list of antibodies can be found in Table S1.

### Intracellular flow cytometry detecting human γ-globin expression

Erythroid cells were collected at the end of ED and stained with CD235a (GlyA, ExBio, or Dako), a surface marker of late erythropoiesis. To evaluate the HbF expression, erythroid cells were fixed in 4% paraformaldehyde followed by permeabilization in 1:1 acetone/H_2_O and stained with anti-human γ-globin (51.7, Santa Cruz). Enucleated erythroid cells were measured as the cell fraction negative for NucRed (Thermo Fisher Scientific).

### Cytospin slide preparation

Cytospins of 0.3–0.5 × 10^5^ cells were prepared during erythroid and myeloid differentiation by cytocentrifugation (ROTOFIX 32, Hettich Zentrifugen) at 500 rpm for 5 min. Cytospins were air dried and then stained with Giemsa/May-Grünwald (Merck, Darmstadt, Germany) for 8 and 3 min, respectively.

### HbF immunofluorescence

Slides with RBC cytospins were immersed in ice-cold methanol for 18 min, rinsed with PBS and H_2_O for 2 min each and air-dried. Slides were then incubated with anti-HbF-FITC (Santa Cruz, SC-21756) at a concentration of 20–60 μg/mL in PBS for 90 min in humidified chambers at 37°C. Slides were then intensively rinsed with PBS and mounting Vectashield. Photos were taken with an EVOS M5000 fluorescent microscope (Invitrogen).

### qRT-PCR

Total RNA was extracted from differentiated CD235a^+^ cells (day 18 of ED culture) using the RNeasy Mini Kit (QIAGEN, cat. no. 74104), following manufacturer’s instructions. A Quantitect reverse transcription kit (QIAGEN, cat. no. 205311) and power SYBRTM green PCR master mix (Thermo Fisher Scientific, cat. no. 4367659) were used. Real-time qPCR was performed on a StepOnePlus Real-Time PCR System (Applied Biosystems). The following primer pairs were used in this work: huActin-b forward, 5′-CCTGCAGAGTTCCAAAGGAGA-3′, and reverse, 5′-AGAAAATCTGGCACCACACC-3ʹ; human γ-globin forward, 5′-GTGGA AGATGCTGGAGGAGAAA-3′, and reverse, 5′-TGCCATGTGCCTTGACTTTG-3ʹ; human β-globin forward, 5′-GGTGCCCTTGAGGTTGTC-3′, and reverse, 5′-ATGAAGTTGGTGGTGAGGC-3ʹ.

### Measurement of base conversion by Sanger sequencing and NGS

Genomic DNA was isolated from cells during several time points of the differentiation cultures, as well as from MACS-isolated cells from the chimeric bone marrow, using a DNeasy Blood&Tissue Kit (QIAGEN, cat. no. 69506) according to the manufacturer’s instructions. For Sanger sequencing, genomic segments encompassing the target sites were amplified using specific primers. For the HBG: forward, 5′-GACGTTCCAGAAGCGAGTGT-3′, reverse, 5′-CCCTTCCCCACACTATCTCA-3ʹ. For the CD33 site: forward, 5′-CCACTCCCTTCCTCTTTTCTGCTCAC-3′, reverse, 5′-GTCCCTGGATATAATGGCTCCTTCC-3ʹ. The amplicons were purified using a QIAquick PCR Purification Kit (QIAGEN, cat. no. 28106) and sequenced using the forward primer for each site. The base editing level was quantified based on Sanger sequencing (Eurofins Genomics) results by using EditR1.0.10.

For NGS, the HBG1/2 target site was amplified using the following primers: HBG-NGS forward, 5′-**ACACTCTTTCCCTACACGACGCTCTTCCGATCT**GACGTTCCAGAAGCGAGTGT-3′, reverse, 5′-**GACTGGAGTTCAGACGTGTGCTCTTCCGATCT**CCCTTCCCCACACTATCTCA-3ʹ; CD33-NGS forward 5′- **ACACTCTTTCCCTAC ACGACGCTCTTCCGATCT**CCACTCCCTTCCTCTTTTCTGCTCAC-3′ and reverse 5′- **GACTGGAGTTCAGACGTGTGCTCTTCCGATC T**GTCCCTGGATATAATGGCTC CTTCC-3ʹ (the bold letters are the adaptors for library construction). After cleaning up using the QIAquick PCR Purification Kit (QIAGEN, cat. no. 28106), the amplicons were submitted for NGS with Amplicon-EZ by Genewiz (South Plainfield, NJ). At least 200,000 reads per amplicon were acquired to probe the types of mutations. Data were aligned to the HBG1/2 and CD33 reference sequences and analyzed using the CRISPResso2,^32^ a python-based genome-editing analysis tool.

### Animal studies

All experiments involving animals were conducted in accordance with the institutional guidelines set forth by the University of Washington. The University of Washington receives accreditation from the Association for the Assessment and Accreditation of Laboratory Animal Care International (AALAC) and all live animal work conducted at the university is in accordance with the Office of Laboratory Animal Welfare (OLAW) Public Health Assurance (PHS) policy, USDA Animal Welfare Act and Regulations, the Guide for the Care and Use of Laboratory Animals, and the University of Washington’s Institutional Animal Care and Use Committee (IACUC) policies. The studies were approved by the University of Washington IACUC (protocol no. 3108-01).

### Mobilization and *in vivo* editing of CD34^+^ cells in a humanized NBSGW mouse model

The immunodeficient NOD.Cg-KitW-41J Tyr+ PrkdcscidIl2rgtm1Wjl/ThomJ (NBSGW) mice were obtained from The Jackson Laboratory (Bar Harbor, ME). NBSGW mice have a low life expectancy, and in our facility they became sick after the age of 4 months, which limited the total length of our studies. A humanized model was generated by transplanting CD34^+^ cells from healthy donors into NBSGW mice (1 × 10^6^/recipient), post partial myeloablation (TBI 100 rad). Six weeks post transplantation, the mice, having a human bone marrow chimerism, were mobilized by a 7-day mobilization scheme, including G-CSF 250 μg/kg i.p. (days 1–6) and AMD3100 5 mg/kg i.p. (days 5–7), as described previously.^33^ Forty minutes post last AMD3100 injection, mice received an i.v. injection the HDAd-ABE8e-sgHBG-sgCD33 vector at a total dose of 8 × 10^10^ viral particles (divided in two doses, 30 min apart). Sixteen and 2 h before i.v. injection of HDAd-ABE8e-sgHBG-sgCD33 vector, the animals received dexamethasone (i.p., 10 mg/kg). One week post *in vivo* transduction, the animals injected i.p. with freshly prepared GO (1 mg/kg). Two months post *in vivo* selection, NBSGW mice were sacrificed, and bone marrow cells were collected for assessment of multilineage engraftment and HbF expression. Human CD235a^+^ and CD45^+^ cells were isolated from the bone marrow and analyzed for editing levels.

To assess the multilineage engraftment of CD34^+^ cells in the bone marrow of NBSGW mice post transplantation, the following antibodies were used: CD45-APC (BD Biosciences), CD19-PerCP (BioLegend), CD3-FITC (BioLegend), CD33-PE (BD Biosciences), and CD235a-PE (BD Biosciences). To measure the percentage of human HSPCs in peripheral blood of NBSGW mice post mobilization, the following antibodies were used: CD45-APC (BD Biosciences, San Jose, CA) and CD34-PE (BioLegend). A list of antibodies used in this study is shown in Table S1.

### MACS

The human CD235a^+^ cells from chimeric bone marrow were isolated using a CD235a-PE antibody and anti-PE microbeads (Miltenyi Biotec, San Diego, CA) according to the manufacturer’s instructions. The human CD45^+^ cells from chimeric bone marrow were isolated using human CD45 microbeads (cat. no. 130-045-801) (Miltenyi Biotec) according to the manufacturer’s instructions. The positive fractions were used for analysis of editing rates in HBG and CD33 sites. The purity of cells (hCD235a^+^ or hCD45^+^) was measured via flow cytometry before DNA extraction. If the purity was less than 80%, a second column was used to increase purity to >95%.

### CFU cultures

Totals of 2,000–3,000 CD34^+^ cells or 5 × 10^4^ hCD45^+^ cells isolated from chimeric bone marrow were plated in semi-solid methylcellulose-based medium containing cytokines, MethoCult H4434 (STEMCELL Technologies), according to the manufacturer’s instructions. After 2 weeks of incubation, CFUs were classified and enumerated under a light microscope by trained operators.

### Blood analysis

Blood samples were collected into EDTA-coated tubes, and analysis was performed on a HemaVet 950FS (Drew Scientific, Waterbury, CT).

### Statistics

Data are presented as mean ± SEM. For comparisons of multiple groups, two-way ANOVA with Bonferroni post hoc testing was employed. Student’s t test was used for comparisons between two groups. Statistical analysis was performed using GraphPad Prism version 10.0.3 (GraphPad Software). A *p* value less than 0.05 was considered significant.

## Data and code availability

All data necessary to interpret, verify and extend the research in the article will be made available.

## Acknowledgments

The study was supported by 10.13039/100000002NIH grants R01HL128288 and R01HL141781 (to A.L.), by a grant from Ensoma Bio (to A.L. and H.-P.K.), and by a grant from the 10.13039/100000865Bill and Melinda Gates Foundation (BMGF): INV-017692 (to A.L.). Under the grant conditions of the BMGF, a Creative Commons Attribution 4.0 Generic License has already been assigned to the Author Accepted Manuscript version that might arise from this submission. We thank Theo Koob, Lishan Huang, and Anna Anderson for technical assistance.

## Author contributions

A.L. provided the conceptual framework for the study. A.G. and C.L. designed the experiments. A.G., C.L., and A.L. performed the experiments. H.-P.K. provided critical comments on the manuscript. A.G. and A.L. wrote the manuscript.

## Declaration of interests

A.L. is an academic co-founder of Ensoma Bio without payment.

## Supplemental information

Document S1. Figures S1–S7 and Table S1

Document S2. Article plus supplemental information
