# Supplementary material for: In vitro and in vivo expansion of CD33/HBG promoter-edited HSPCs with Mylotarg
Source: Mol Ther Methods Clin Dev. 2024 Sep 21;32(4):101343. doi: 10.1016/j.omtm.2024.101343 (PMC11490927; doi:10.1016/j.omtm.2024.101343)
Supplement: Document S1. Figures S1–S7 and Table S1 [file mmc1.pdf]

OMTM, Volume *32*

Supplemental information

In vitro and *in vivo* expansion of CD33/HBG promoter-edited HSPCs with Mylotarg

Aphrodite Georgakopoulou, Chang Li, Hans-Peter Kiem, and André Lieber

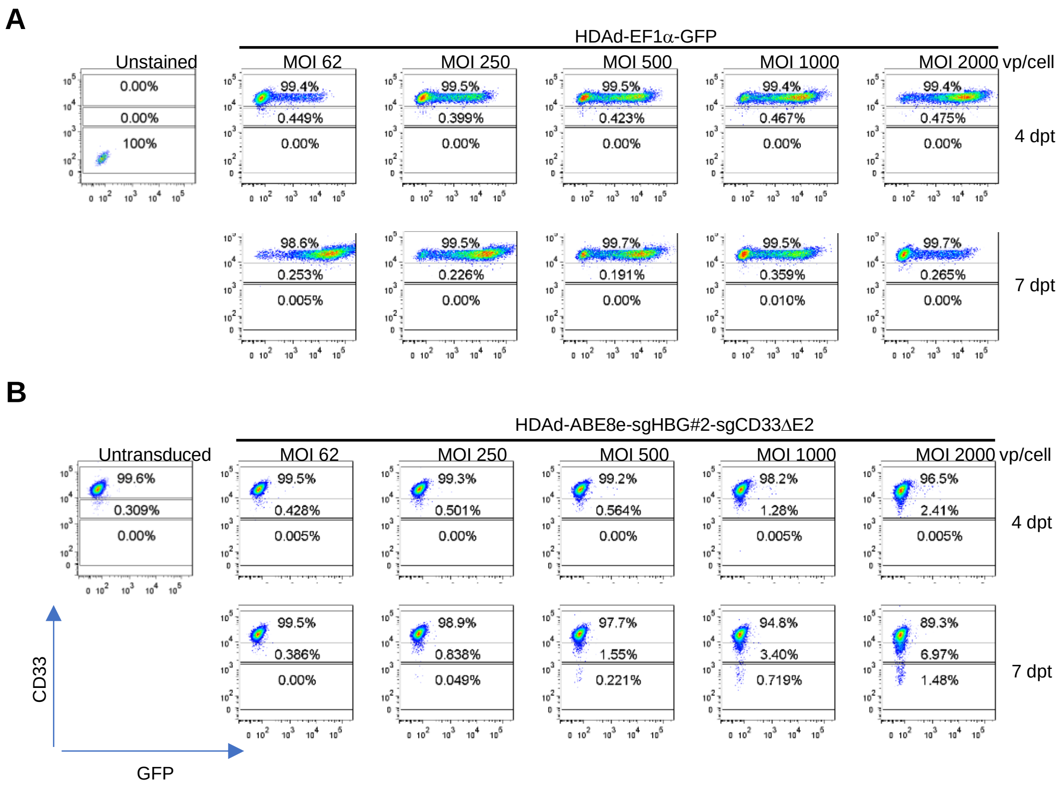

Fig.S1 Control settings for the transduction/GO selection study in ML-1 cells (see Fig.1). A) Transduction of ML-1 cells with a reporter vector expressing GFP (HDAd-EF1α-GFP) at increasing MOIs measured at days 4 and 7 post transduction. The data show efficient transduction at MOIs as low as 62 vp/cell. **B)** Effect of HDAd-ABE8e-sgHBG#2-sgCD33$^{\Delta E2}$ transduction on CD33 expression without GO selection. At day 7 after transduction at an MOI of 2000 vp/cell, the percentage of CD33^{medium} and CD33$^{\Delta E2}$ cells was only 6.97% and 1.48%, respectively.

A

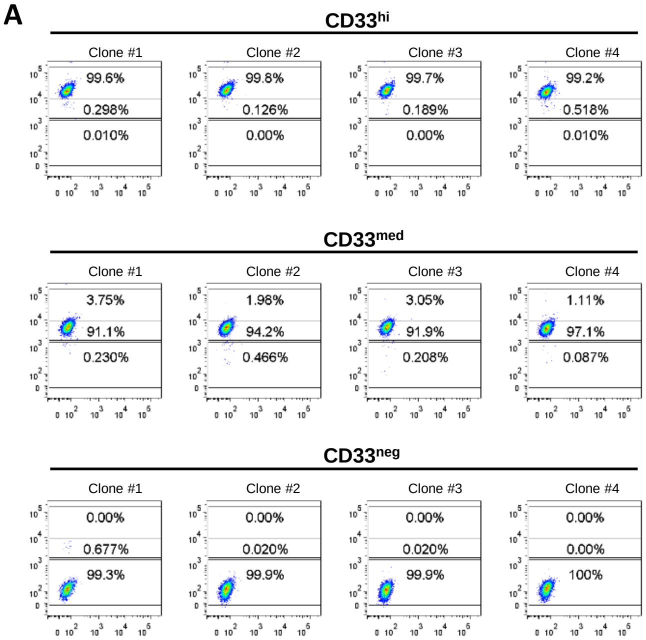

B

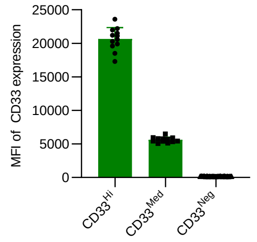

C

CD33hi clones	A_5>G	A_7>G
#1	0	0
#2	0	0
#3	0	0
#4	0	22
#5	0	0
#6	30	0
#7	0	0
#8	0	0
#9	0	0
#10	0	0
#11	0	0
#12	0	0

CD33med clones	A_5>G	A_7>G
#1	59	100
#2	26	81
#3	59	100
#4	81	99
#5	81	100
#6	52	99
#7	80	99
#8	82	100
#9	83	99
#10	81	99
#11	81	100
#12	84	99

CD33neg clones	A_5>G	A_7>G
#1	100	100
#2	100	100
#3	78	100
#4	28	100
#5	100	100
#6	100	100
#7	100	100
#8	76	100
#9	100	100
#10	100	100
#11	80	100
#12	54	100
#13	77	100
#14	100	100
#15	48	100
#16	79	100
#17	49	100
#18	56	100
#19	100	100
#20	75	100
#21	100	100
#22	79	100
#23	100	100
#24	100	100

Fig.S2 Analysis of single-cell derived clones. A) Representative flow plots for $CD33^{hi}$, $CD33^{med}$, $CD33^{neg}$ were analyzed. Shown are the flow plots for 4 clones from each group. **B)** Summary of CD33 expression in ML-1 clones. **C)** Editing rate at the CD33 splicing site. Percent conversion of A5 and A7 adenines. A7>G is the desired target site modification. 100% editing rate at this site is considered bi-allelic. Lower editing rates are considered mono-allelic. Editing rates of less than 30% can be NGS artifacts and are taken as absence of editing.

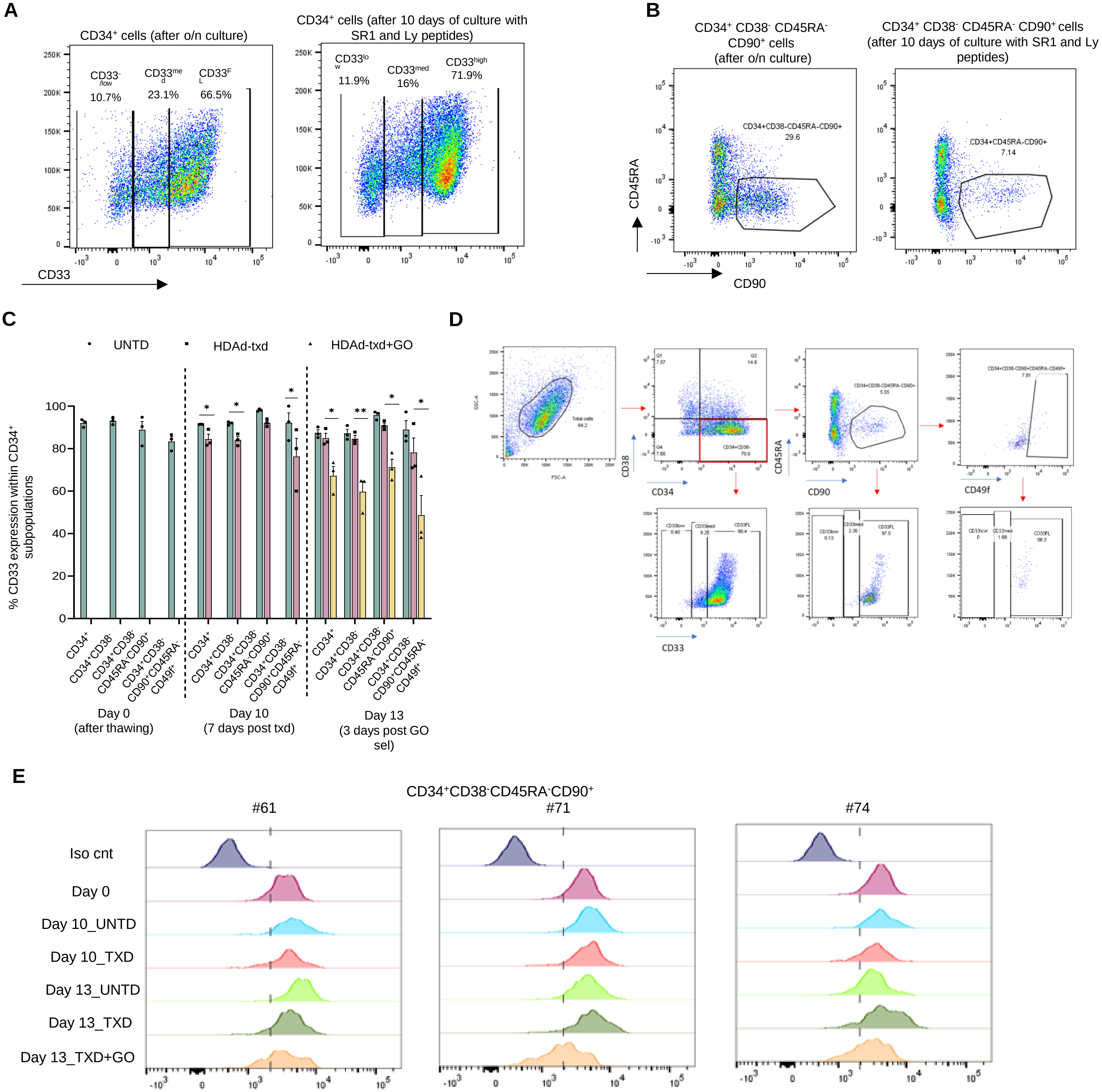

Fig.S3 CD33 expression in human HSPCs. A) CD34⁺ cells from peripheral blood of a G-CSF-mobilized donor. Cryopreserved cells were thawed and cultured o/n or for 10 days in the presence of a peptide cocktail that suppresses differentiation and stained with anti-CD33-antibody. Flow cytometry plots show that the majority of $CD34^+$ cells express CD33 at both time points. **B)** Immunophenotype of $CD34^+$ cells during culture. Flow cytometry plots show the preservation of a more primitive subpopulation of HSCs ($CD34^+/CD45RA^-/CD90^+$ cells) after 10 days in culture. **C)** CD33 expression of HSPC subpopulations on day 0, day 10, and 3-days post GO-selection (day 13). N=3. **D)** Gating strategy for the analysis of phenotype and CD33 expression. Each symbol represents an individual donor. Data are shown as means ± SEM. **p≤0.01, *p≤0.05 (Two-way ANOVA with Bonferroni correction). **E)** CD33 expression in human HSPCs**.** Histograms from three representative donors. CD33 expression is shown within $CD34^+CD38^+CD45RA^-CD90^+$.

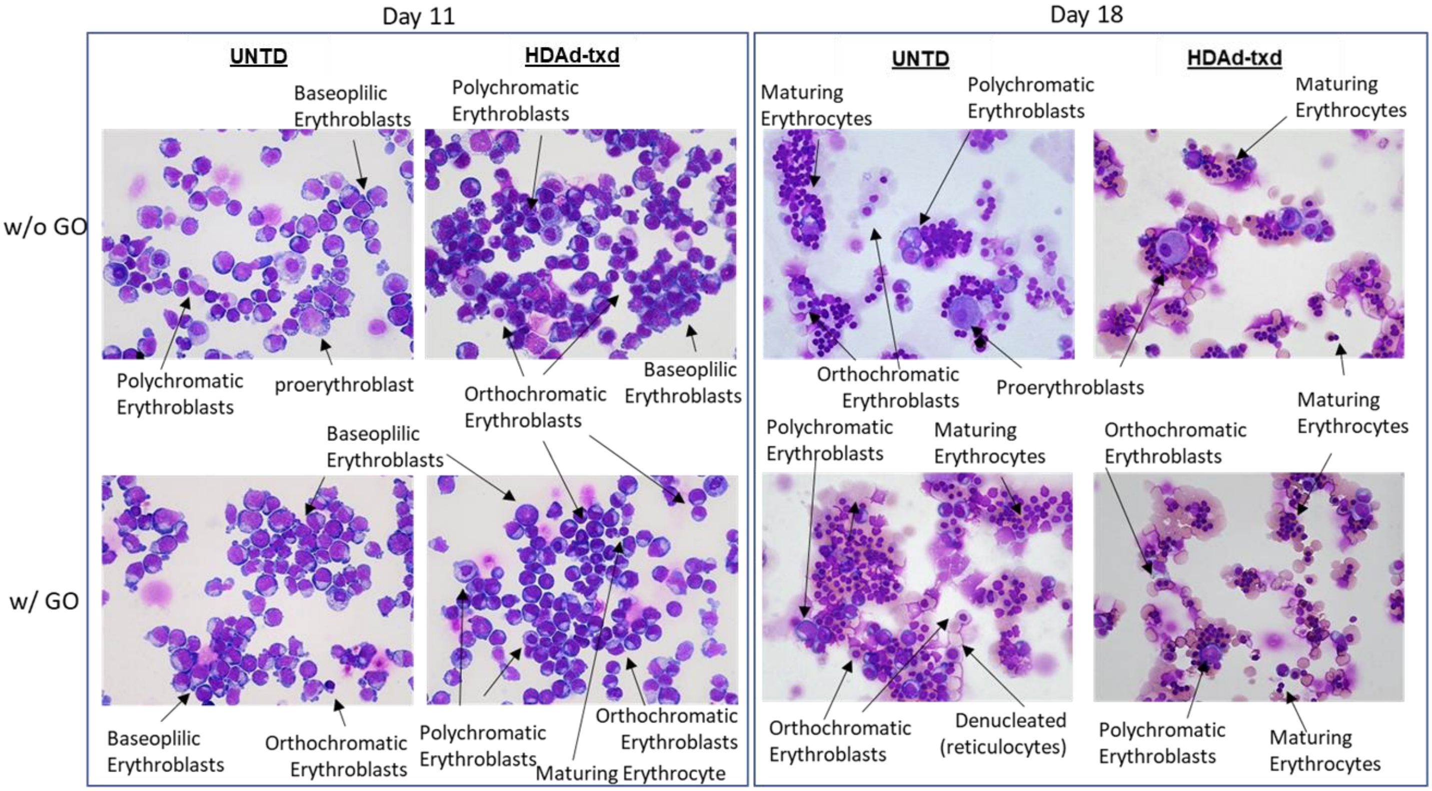

Fig.S4 Morphology of CD34$^+$ cells during erythroid differentiation. Representative images of untransduced, untransduced + GO, transduced and transduced + GO cells on day 11 and 18 of erythroid culture. Higher maturation rates were observed in the HDAd-transduced/edited and GO-treated cells. Hematoxylin & eosin stain; magnification X40. Arrows label cells at different stages of maturation. Proerythroblasts → basophilic erythroblasts → polychromatic erythroblasts → orthochromatic erythroblasts → reticulocytes → erythrocytes.

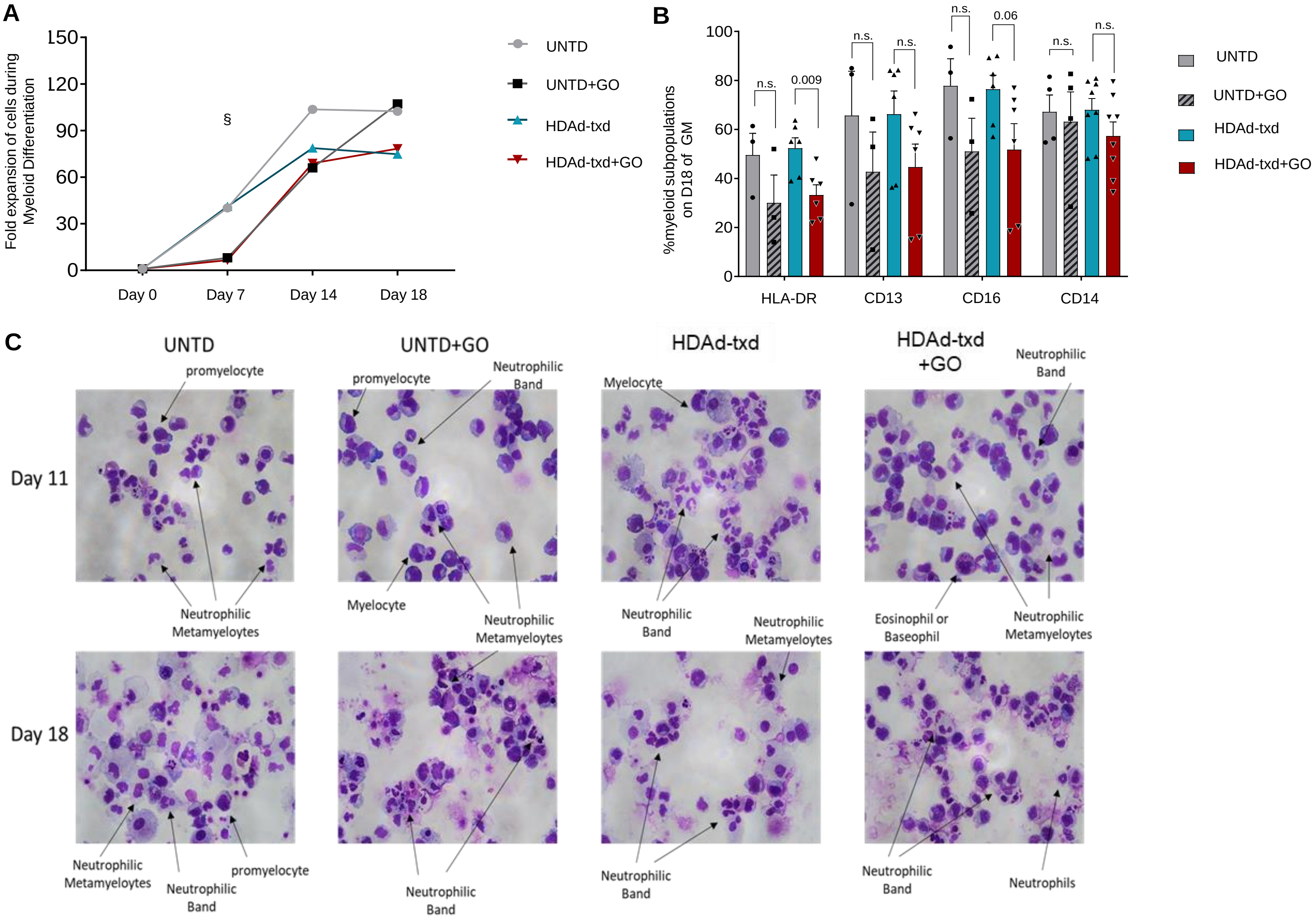

Fig.S5 Expansion of CD34⁺ cells with and without GO-selection in myeloid differentiation medium. A) Proliferation of myeloid cells during culture, presented as fold expansion over time.§ UNTD vs UNTD+GO *p=0.04, TXD vs TXD+GO ***p=0.0003, UNTD vs TXD+GO *p=0.03, TXD vs UNTD+GO ***p=0.0004 (Two-way ANOVA with Bonferroni correction). **B)** Percentage of different myeloid subpopulations during the myeloid culture. Shown is the percentage of HLA-DR$^+$ (monocytes, macrophages, dendritic cells), CD13$^+$ (granulocytes, monocytes), CD16$^+$ (neutrophils), and CD14$^+$ (monocytes) cells on day 18 of differentiation. All plots represent data from at least 4 different donors. Data are shown as means ± SEM. Unpaired t-test. **C)** Representative image of cytospins from day 11 and day 18 of myeloid culture did not show differences in the differentiation and maturation of myeloid cells between the different groups. Hematoxylin & eosin stain; magnification X40. Arrows label cells at different stages of maturation. Blast → promyelocyte → myelocyte → metamyelocyte → neutrophilic band → neutrophil.

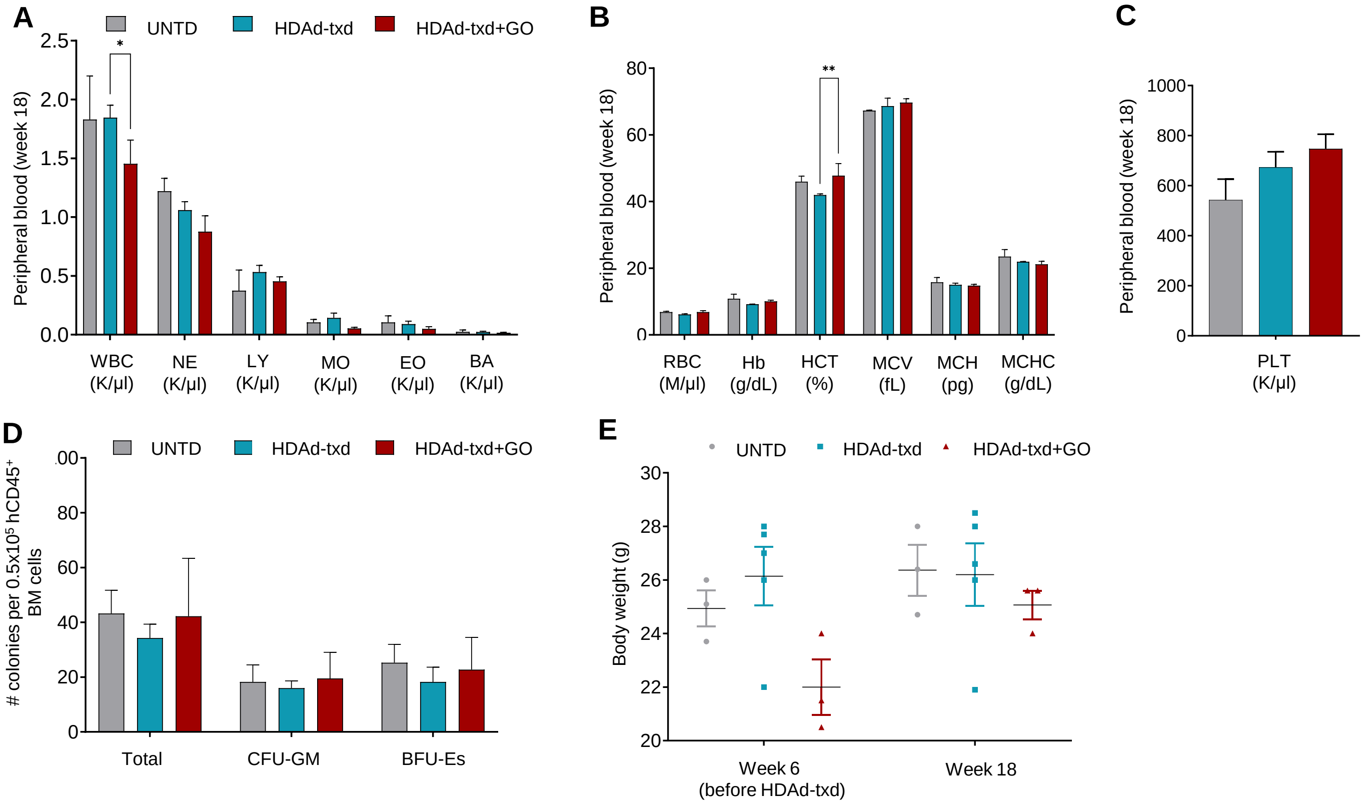

Fig.S6 Safety of *in vivo* administration of GO. A-C) Hematological parameters of *in vivo* transduced NBSGW mice which received GO (N=3) as compared to their untreated counterparts; untransduced (n=2) and *in vivo* transduced without GO- selection (n=5), two months post *in vivo* transduction/selection; **A)** WBC counts **B)** RBC analysis **C)** Platelet counts. **D)** Number of total colonies, BFU-Es and CFU-GM, generated from engrafted human cells in the chimeric BM. **E)** Animal weight, before and after *in vivo* administration of GO. WBC, white blood cells; NE, neutrophils; LY, lymphocytes; MO, monocytes; EO, eosinophils; BA, basophils; RBC, red blood cells; Hb, hemoglobin; HCT, hematocrit; MCV, mean corpuscular volume; MCH, mean corpuscular hemoglobin; MCHC, mean corpuscular hemoglobin concentration; PLT, platelets. Each symbol represents an individual mouse. Data are shown as means ± SEM. **$p \leq 0.01$, *$p \leq 0.05$ (Two-way ANOVA with Bonferroni correction).

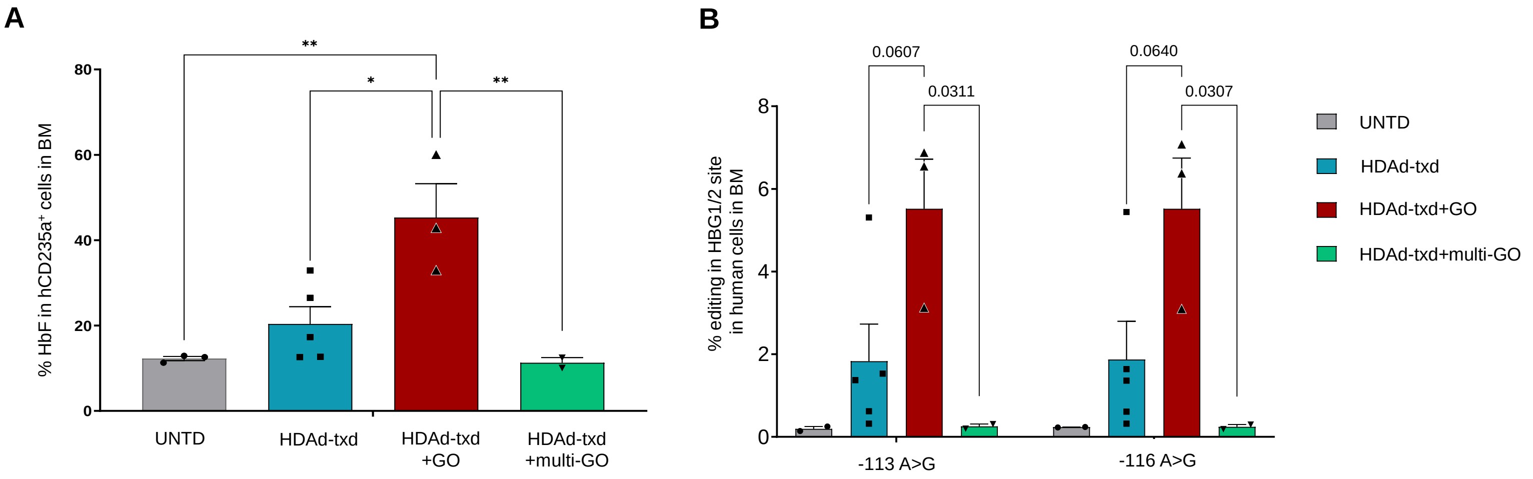

Fig. S7 Multiple rounds of *in vivo* administration of GO in *in vivo* transduced NBSGW mice. A) HbF expression in engrafted human erythrocytes in BM. **B)** Editing levels of the targeted and bystander adenines in the HBG site in human cells from the chimeric BM. Each symbol represents an individual mouse. Data are shown as means ± SEM. **p≤0.01, *p≤0.05 (Two-way ANOVA with Bonferroni correction).

Table S1. List of antibodies used for flow cytometry.

Epitope	Clone	Company	Catalog Number
CD33	P67.6	BD Biosciences	3058564
CD33	9G2	BD Biosciences	555626
CD34	561_APC	BioLegend	343608
CD34	561_PE	BioLegend	343606
CD38	HIT2	BD Biosciences	555460
CD90	5E10	BD Biosciences	555597
CD235a	GA-R2	BD Biosciences	555570
CD71	M-A712	BD Biosciences	555536
CD14	M5E2	BD Biosciences	555397
HLA-DR	L243	BioLegend	307610
CD13	WM15	BioLegend	301706
CD16	B73.1	BioLegend	360720
CD45	HI30	BD Biosciences	555485
CD19	HIB19	BioLegend	302228
CD3	UCHT1	BioLegend	300406
